# Updating the evidence on drugs to treat overactive bladder: a systematic review

**DOI:** 10.1007/s00192-019-04022-8

**Published:** 2019-07-25

**Authors:** Frances C. Hsu, Chandler E. Weeks, Shelley S. Selph, Ian Blazina, Rebecca S. Holmes, Marian S. McDonagh

**Affiliations:** 1 Department of Medical Informatics and Clinical Epidemiology, Oregon Health & Science University, Portland, OR USA; 2 Pacific Northwest Evidence-based Practice Center, Portland, OR USA

**Keywords:** Overactive bladder, Urgency urinary incontinence, Mirabegron, Antimuscarinics, Systematic review, Meta-analysis

## Abstract

**Introduction:**

Overactive bladder (OAB) is a common condition, increasing with age and affecting quality of life. While numerous OAB drugs are available, persistence is low. We evaluated evidence published since 2012 to determine if newer drugs provided better efficacy and harm profiles.

**Methods:**

We searched MEDLINE and the Cochrane Library from 2012 to September 2018 using terms for included drugs and requested information from manufacturers of included drugs. We performed dual review of all systematic review processes, evaluated study quality, and conducted meta-analyses using random effects models.

**Results:**

In addition to 31 older studies, we included 20 trials published since 2012 (*N* = 16,478; 4 good, 11 fair, and 5 poor quality). Where statistical differences were found, they were clinically small (reductions of < 0.5 episodes/day). Solifenacin plus mirabegron improved efficacy outcomes over monotherapy with either drug, but significantly increased constipation compared with solifenacin and dry mouth compared with mirabegron. Solifenacin reduced incontinence over mirabegron and tolterodine and urgency episodes over tolterodine. Mirabegron did not differ from tolterodine in efficacy but had significantly lower incidence of dry mouth than solifenacin or tolterodine. Fesoterodine showed significant improvements but also anticholinergic effects vs. tolterodine. Oxybutynin, solifenacin, and tolterodine had similar efficacy, but dry mouth led to greater discontinuation with oxybutynin. Blurred vision, cardiac arrhythmia, and dizziness were uncommon.

**Conclusion:**

New evidence confirms small, but clinically uncertain, differences among monotherapies and also between combination and monotherapy, regardless of statistical significance. While drugs mainly differed in incidence of dry mouth or constipation, none provided improved efficacy without increased harms.

**Electronic supplementary material:**

The online version of this article (10.1007/s00192-019-04022-8) contains supplementary material, which is available to authorized users.

## Introduction

Overactive bladder (OAB) is defined by the International Continence Society as a syndrome of urinary urgency, often with urinary frequency and nocturia, in the absence of local pathological factors [1]. OAB differs from urinary incontinence, though the two are not mutually exclusive. A subset of patients with OAB complains of urgency urinary incontinence (i.e., involuntary leakage accompanied by or immediately preceded by urgency; UUI) and/or stress incontinence (i.e., inability to retain urine when sneezing or coughing), especially women [2, 3]. Men with OAB symptoms often have comorbid bladder outlet obstruction secondary to benign prostatic hyperplasia [4].

Overactive bladder is a common syndrome, and prevalence increases with age. In adults, prevalence ranges from 7 to 27% in men and 9–43% in women [5]. Risk factors for OAB include smoking, obesity, arthritis, depression, heart disease, and irritable bowel syndrome [6]. Additional risk factors specific for men include race (higher in non-White men), increasing age, and a history of prostate disease; additional risk factors for women include neurological conditions (e.g., multiple sclerosis), diabetes, pregnancy, urinary tract infection, uterine prolapse, hysterectomy, and menopause [6]. Symptoms associated with OAB have a significant negative impact on affected individuals’ lives. Numerous studies (e.g., EPIC, NOBLE, EpiLUTS) found that individuals with OAB have significantly reduced quality of life compared with individuals without OAB, regardless of incontinence status [7–10]. Using validated patient-reported outcome assessments, patients with OAB reported significantly higher levels of anxiety and depression, reduced general health status, and poorer sleep quality compared with controls. Moreover, OAB symptoms interfered with individual’s ability to perform self-care and work activities [9, 11].

The American Urological Association and the Society of Urodynamics, Female Pelvic Medicine & Urogenital Reconstruction updated a clinical practice guideline on treatment of non-neurogenic overactive bladder in 2015 [5]. The guideline recommended behavioral therapies, such as bladder training and control strategies, pelvic floor muscle training, and fluid management, as first-line therapy. Second-line treatments included antimuscarinic drugs and β3-adrenoceptor agonists, reserving onabotulinumtoxinA injections, peripheral tibial nerve stimulation, and sacral neuromodulation as third-line treatments. Detrusor muscle contractility is primarily controlled by the parasympathetic nervous system via acetylcholine acting on muscarinic receptors [12]. Antimuscarinic drugs block acetylcholine from binding to muscarinic receptors. Antimuscarinic drugs are associated with systematic anticholinergic adverse effects, including dry mouth and constipation, with varying incidences. These and other adverse effects contributed to low medication persistence [13]. Muscarinic receptor subtypes M_2_ and M_3_, targeted by solifenacin, are the predominant subtypes in the detrusor muscle but also present in other tissues such as the gut [12]. The M_2_ subtype works in conjunction with β_3_ receptors to encourage smooth muscle relaxation [14]. The M_3_ subtype is selectively targeted by darifenacin [15]. In contrast, mirabegron mimics sympathetic activity by stimulating the β3-adrenoceptors on the detrusor muscle, promoting bladder relaxation during the filling stage [16]. Increased blood pressure, nasopharyngitis, urinary tract infection, and headache are common adverse effects with mirabegron, and dizziness and urinary retention have also been reported [17].

Although numerous drugs (primarily antimuscarinic drugs) have been approved by the Food and Drug Administration (FDA) to treat OAB, persistence with pharmacotherapy is generally poor. In a 1-year study based on the UK General Practice longitudinal prescription database, < 25% of patients remained on the initially prescribed drug [18]. Patients remained on mirabegron for a median of 101 days and about 55 days for fesoterodine, solifenacin, tolterodine extended release (ER), and trospium [18]. The reasons for poor persistence are complex, including inadequate efficacy, adverse effects, age, cost or insurance coverage, interactions with drugs and comorbidities, and combinations of these [19]. Selecting drug treatment with the best net benefit may improve persistence with treatment and ultimately quality of life for a given patient.

A Cochrane Review published in 2012 compared anticholinergic drugs for OAB [20]. However, it did not include the β3-adrenoceptor stimulator, mirabegron. With the FDA approval of mirabegron and a combination product with solifenacin for OAB, we sought to update this evidence. We conducted a systematic review and meta-analyses comparing the effectiveness of drugs approved by the FDA to treat OAB symptoms.

## Materials and methods

This work was originally conducted for the Drug Effectiveness Review Project (DERP), a collaboration of state Medicaid agencies that commission systematic reviews of drug therapies for use in policymaking. The scope of the original review was determined in consultation with DERP participants, and a protocol was developed a priori. The work presented here is an update of that work with addition of the combination product. The systematic review was conducted according to the methods developed specifically for DERP [21] and are in accordance with methods established by the US Agency for Healthcare Research and Quality (AHRQ) for the Evidence-based Practice Center (EPC) program [22].

### Eligibility criteria

Studies of adults with symptoms of OAB, including UUI and mixed incontinence, were included. Studies of patients with only stress incontinence or neurogenic detrusor overactivity were excluded. We included the 2012 Cochrane Review of anticholinergic drugs as the baseline evidence [20] and randomized controlled trials (RCTs) published since the review that compared any formulation of darifenacin, fesoterodine, mirabegron, oxybutynin, solifenacin, tolterodine, trospium, and a combination of mirabegron with solifenacin, with each other. We only included study arms with doses approved by the FDA.

### Search strategy and study selection

To identify relevant citations, we searched MEDLINE, MEDLINE In-Process, the Cochrane Central Register of Controlled Trials, and the Cochrane Database of Systematic Reviews in September 2018 using terms for included drugs (see Table S1 for complete search strategy). We limited the electronic searches to publications in English and excluded studies tagged as “animal.” We also searched FDA’s Center for Drug Evaluation and Research for medical reviews, www.ClinicalTrials.gov, and requested information on published and unpublished studies (or study data) from manufacturers of included drugs. Titles and abstracts of citations identified through literature searches were assessed for inclusion by one reviewer, and a second reviewer checked all citations excluded by the first reviewer. Full-text articles of potentially relevant citations were retrieved and assessed for inclusion independently by two reviewers. Disagreements were resolved by consensus.

### Data extraction and study assessment

We abstracted information on baseline population characteristics, interventions, subject enrollment and discontinuation, and results for effectiveness and harms outcomes. Efficacy outcomes examined included change from baseline number of incontinence and urgency (grade 3 or 4) episodes, micturition frequency, proportion of patients reporting no incontinence over 3 days at end of study, and patient-reported symptom assessment using the Patient Perception of Bladder Condition (PPBC), Overactive Bladder Questionnaire (OAB-q) Symptom Bother score, or the Overactive Bladder Symptom Score (OABSS). The PPBC contains six items assessing change scores from −2 to 2 with negative scores indicating improvement. Clinically meaningful difference has not yet been established for the PPBC [23]. The OAB-q Symptom Bother score contains eight items on a 100-point scale with a suggested minimal clinically important difference (MCID) of ten points [24]. The OABSS contains four questions, one each assessing daytime frequency, nighttime frequency, urgency, and UUI with a maximum score of 2, 3, 5, and 5, respectively (higher score indicates worse symptom), yielding a total possible score of 15 [25]. An MCID of 3 points has been suggested [25]. Harm outcomes examined included withdrawals due to adverse events, serious adverse events (SAEs) as defined by individual studies, blurred vision, constipation, dizziness, dry mouth, QT prolongation, arrhythmia, and other cardiac outcomes. Data abstraction was performed by one reviewer and checked by a second reviewer. We assessed the internal validity (quality) of trials based on the predefined DERP criteria [21], including method used for randomization, allocation concealment, similarity of compared groups at baseline, loss to follow-up, and the use of intent-to-treat analysis. Studies with a high risk of bias were rated poor quality, trials that met all criteria were rated good quality, and the remainder were rated as fair. Two reviewers independently assessed the quality of each study and differences were resolved by consensus.

### Data synthesis

We evaluated the results by examining the clinical and methodological characteristics of the included studies, exploring relationships in the data, and describing patterns across studies in the direction and magnitude of effects. Poor-quality studies were not synthesized with the rest of the evidence. Applicability of the evidence was described using the PICOTS (population, intervention, comparator, outcomes, timing, setting) framework. When outcomes were not reported as relative risk (RR) or odds ratio but provided sufficient data, we calculated these ratios. Meta-analysis was conducted when a sufficient number of studies investigated the same drug dose and were homogeneous enough to justify combining their results. We evaluated outcomes in terms of change from baseline rather than a comparison of end point scores as was done in the prior review [20]. We imputed variance data where necessary. Random effects models were used to estimate pooled effects using pairwise meta-analyses [26]. The I^2^ statistic (the proportion of variation in study estimates due to heterogeneity) was calculated to assess heterogeneity across studies [27, 28]. Potential sources of heterogeneity were examined with subgroup analysis by factors such as study design, study quality, variations in interventions, and patient population characteristics. Meta-analyses were conducted using STATA 10.1 (StataCorp, College Station, TX). Where eight or more studies of a given intervention comparison and outcome were available, publication bias was assessed using funnel plots.

## Results

**Fig. 1:**
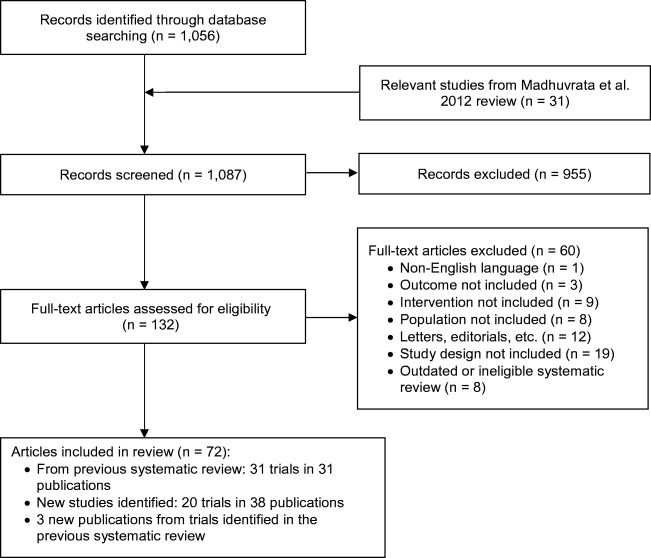
PRISMA (Preferred Reporting Items for Systematic Reviews and Meta-Analyses) flow diagram

In this systematic review update, we identified 20 new RCTs (in 38 records and 3 new publications for trials identified in the previous systematic review) [29–69] published since the 31 RCTs included in the 2012 review [20]. See Fig. 1 for the PRISMA flow diagram (see Table S2 for list of included studies). We also included the full publication and data from an RCT [33] comparing darifenacin vs. solifenacin that was only published as an abstract at the time of the 2012 review. This study and five others (in six records) were rated poor quality because of unclear allocation concealment, blinding, and issues related to missing data and were not discussed in this report [31, 36, 45, 49, 50, 64]. Five RCTs were rated good quality and 10 were fair quality. Since mirabegron was approved after the 2012 review, all evidence involving mirabegron is new (14 RCTs total). Two new comparisons added were fesoterodine vs. solifenacin [39] and darifenacin vs. trospium [52]. Excluded studies with reasons for exclusion are listed in Table S3. Detailed study characteristics and quality assessments of included studies are in Tables S4 and S5.

Table 1 shows characteristics of new included studies. Considering fair- and good-quality RCTs identified in this update, trial sample size of included populations ranged from 60 to 3080. Trial durations ranged from 4 to 52 weeks (median 12 weeks). Of 12 trials that reported funding source, all but one were funded by industry. Mean age of patients was 57.4 (SD 13.4) years with a mean BMI of 28.4. Similar to RCTs included in the 2012 review, patients were predominantly female (77.7%) and White (89.7%). UUI was the most common form of OAB (58.6%), followed by mixed incontinence (22.2%) then OAB without incontinence (19.4%). Patients experienced mean 67.4 months of OAB symptoms and 59.2% had previous pharmacotherapy for OAB. At baseline, patients reported means of 3 incontinence episodes, 6.1 urgency episodes, and 2.5 UUI episodes per day. As with previously included RCTs, there was a wide range of baseline incontinence episodes per day (range 1.9 to 8.9 episodes per day, median 2.78). Baseline numbers of urgency and UUI episodes were more consistent, with a range of 4.2 to 8.2 urgency episodes and 1.7 to 3.9 UUI episodes per day.

**Table 1:** Baseline characteristics of studies published after 2012 review

Study (trial name)	Country	Interventions	Sample size (selected sample size)	Duration, weeks	Mean age, years (SD)	Baseline type(s) of OAB, %	Prior drug treatment, %	Quality rating
Mirabegron vs. solifenacin								
Abrams et al., 2015 (SYMPHONY) [29, 58]	Multinational	Mirabegron 25 mg Mirabegron 50 mg Solifenacin 5 mg Solifenacin 10 mg Solifenacin 5 mg + mirabegron 25 mg Solifenacin 5 mg + mirabegron 50 mg	*N* = 1306 (*n* = 686)	12	54.5 (19.1)	UUI: 25.7 M: 13.4 F: 60	46.9%	Fair
Batista et al., 2015 (BEYOND) [32, 59]	Multinational	Mirabegron 50 mg Solifenacin 5 mg	*N* = 1887	12	57.0 (13.9)	UUI: 41.2 M: 15.7 F: 44.2	100%	Good
Drake et al., 2016 Drake et al., 2017 (BESIDE) [37, 38, 60]	Multinational	Solifenacin 5 mg + mirabegron 25–50 mg Solifenacin 5 mg Solifenacin 10 mg	*N* = 2174	12	57.5 (13.3)	UUI: 100 M: 0 F: 0	68.2%	Good
Gratzke et al., 2018 (SYNERGY II) [40, 62]	Multinational	Mirabegron 50 mg Solifenacin 5 mg + mirabegron 50 mg Solifenacin 5 mg	*N* = 1829	52	58.5 (13.0)	UUI: 71.5 M: 28.4 F: NR	53.5%	Good
Herschorn et al., 2017 Robinson et al., 2017 White et al., 2018 (SYNERGY) [41, 61, 65, 68]	Multinational	Mirabegron 25 mg Mirabegron 50 mg Solifenacin 5 mg Solifenacin 5 mg + mirabegron 25 mg Solifenacin 5 mg + mirabegron 50 mg	*N* = 3527 (*n* = 3080)	12	57.1 (13.5)	UUI: 65.3 M: 34.7 F: NR	46.2%	Fair
Kinjo et al., 2016 [49]	Japan	Mirabegron 25–50 mg Solifenacin 2.5–5 mg	*N* = 148	52	62.5 (10.9)	NR	0%	Poor
Kosilov et al., 2015 [50]	Russia	Mirabegron 50 mg Solifenacin 10 mg Solifenacin 10 mg + mirabegron 50 mg	*N* = 239	6	71.2 (NR)	NR	NR	Poor
Vecchioli Scaldazza & Morosetti, 2016 [67]	Italy	Mirabegron 50 mg Solifenacin 5 mg	*N* = 80	12	57 (NR)	NR	NR	Fair
Mirabegron vs. tolterodine								
Chapple et al., 2013 (TAURUS) [35, 54]	Multinational	Mirabegron 50 mg Tolterodine ER 4 mg	*N* = 2444 (*n* = 1624)	52	59.4 (12.5)	UUI: 37.7 M: 27.2 F: 35.0	59.1%	Fair
Chapple et al., 2013 Astellas, 2010 (DRAGON) [30, 34]	Japan	Mirabegron 25 mg Mirabegron 50 mg Tolterodine ER 4 mg	*N* = 927 (*n* = 423)	12	57.0 (12.4)	UUI: 43.9 M: 26.7 F: 29.4	46.3%	Fair
Khullar et al., 2013 Khullar et al., 2016 (SCORPIO) [47, 48, 55]	Europe, Australia	Mirabegron 50 mg Tolterodine ER 4 mg	*N* = 1987 (*n* = 992)	12	59.1 (12.6)	UUI: 39.5 M: 22.7 F: 37.9	49.7%	Good
Kuo et al., 2015 [51, 57]	Multinational	Mirabegron 50 mg Tolterodine ER 4 mg	*N* = 749	12	54.1 (14.4)	UUI: 38.7 M: 18.6 F: 42.6	52%	Fair
Staskin et al., 2018 Herschorn et al., 2018 (PREFER) [43, 63, 66]	US and Canada	Mirabegron 50 mg Tolterodine ER 4 mg	*N* = 626	8	53.7 (13.7)	UUI: 40.2 M: 34.6 F: 24.7	5% (non-drug only)	Fair
Yamaguchi et al., 2014 [56, 69]	Japan	Mirabegron 50 mg Tolterodine 4 mg	*N* = 1139 (*n* = 758)	12	58.3 (13.8)	UUI: 63.1 M: 27.4 F: 9.5	35.8%	Fair
Solifenacin vs. fesoterodine								
Ercan et al., 2015 [39]	Turkey	Solifenacin 5 mg Fesoterodine 4 mg	N = 119	12	58.5 (10.9)	NR	NR	Fair
Darifenacin vs. trospium								
Manjunatha et al., 2015 [52]	India	Darifenacin 7.5 mg Trospium ER 60 mg	N = 60	4	64.1 (8.6)	NR	NR	Fair
Oxybutynin vs. tolterodine								
Aziminekoo et al., 2014 Jafarabadi et al., 2015 [31, 45]	Iran	Oxybutynin 5 mg Tolterodine 2 mg	*N* = 100	4	53.0 (12.0)	NR	NR	Poor
Jafarabadi et al., 2015 [46]	Iran	Oxybutynin 5 mg Tolterodine 2 mg	N = 301	12	54.7 (9.0)	NR	NR	Fair
Tolterodine vs. solifenacin								
Rana et al., 2016 [64]	Pakistan	Tolterodine 4 mg Solifenacin 5 mg	*N* = 830	12	57.3 (11.5)	NR	NR	Poor
Darifenacin vs. solifenacin								
But et al., 2012 [33]	Slovenia	Darifenacin 7.5 mg Solifenacin 5 mg	*N* = 77	12	54.8 (11.5)	NR	NR	Poor
Trospium vs. oxybutynin vs. tolterodine								
Dede et al., 2013 [36]	Turkey	Trospium 40 mg Oxybutynin 15 mg Tolterodine 4 mg	*N* = 90	6	51.8 (10.5)	NR	NR	Poor

Abbreviations: F, frequency; M, mixed stress/urge incontinence; NR, not reported; SD, standard deviation; UUI, urgency urinary incontinence

### Mirabegron plus solifenacin vs. solifenacin

Four trials (*N* = 6430 for included drug doses) compared the combination of mirabegron (25 to 50 mg) plus solifenacin 5 mg vs. solifenacin 5 mg, with three lasting 12 weeks [29, 37, 41] and one lasting 52 weeks [40]. Two trials were rated good quality and two were fair quality. Table 2 shows change from baseline, pooled mean differences (MD), and risk ratio for efficacy outcomes for each drug comparison. The combination of mirabegron 50 mg with solifenacin 5 mg showed significant improvement on all efficacy outcomes compared with solifenacin 5 mg. However, patients still experienced more than one incontinence and about three urgency episodes per day. Moreover, the absolute difference between combination therapy and monotherapy was less than one episode of incontinence, urgency and micturitions per day (−0.18, −0.58, and − 0.41, respectively). There was moderate heterogeneity (I^2^ = 59%) in the pooled estimate of reduction of urgency episodes, but all trials consistently favored combination therapy. Of patients with incontinence at baseline, significantly more patients on combination therapy reported no incontinence over 3 days at end of treatment (45% vs. 36%). All four trials reported measures of patient-assessed change in symptoms using both the PPBC and the OAB-q Symptom Bother score, and pooled results favored combination therapy on both assessments (Table 3). On the OAB-q Symptom Bother score, both treatment groups achieved the MCID from baseline.

**Table 2:** Efficacy outcomes in short-duration trials (4 to 12 weeks) of pharmacotherapies for overactive bladder

	Incontinence episodes /24 h				3-day 0 incontinence		Urgency episodes /24 h				Micturitions /24 h			
	No. of Studies	Baseline (mean)^a^	Change from baseline	MD (95% CI; I^2^)	No. of studies	RR (95% CI; I^2^)	No. of studies	Baseline (mean)^a^	Change from baseline	MD (95% CI; I^2^)	No. of studies	Baseline (mean)^a^	Change from baseline	MD (95% CI; I^2^)
Mirabegron 50 mg + solifenacin 5 mg vs. solifenacin 5 mg	4	3.17	−1.84 vs. −1.63	**−0.18 (−0.31, −0.05; I**^**2**^ **= 0%)**	3	**1.23 (1.13, 1.34; I**^**2**^ **= 0%)**	4	6.21	−3.34 vs. −2.73	**−0.58 (−0.89, −0.28; I**^**2**^ **= 59%)**	4	10.28	−2.20 vs. −1.69	**−0.41 (−0.54, −0.27; I**^**2**^ **= 0%)**
Mirabegron 50 mg + solifenacin 5 mg vs. mirabegron 50 mg	3	3.14	−1.85 vs. −1.56	**−0.34 (−0.52, −0.16; I**^**2**^ **= 8%)**	2	1.18 (0.98, 1.41; I^2^ = 22%)	3	6.38	−3.47 vs. −2.73	**−0.77 (−1.02, −0.52; I**^**2**^ **= 0%)**	3	10.78	−2.41 vs. −1.94	**−0.56 (−0.75, −0.37; I**^**2**^ **= 0%)**
Mirabegron 50 mg vs. solifenacin 5 mg	4	2.85	−1.51 vs. −1.70	**+0.20 (0.02, 0.38; I**^**2**^ **= 12%)**	3	1.01 (0.93, 1.09; I^2^ = 0%)	4	6.75	−3.76 vs. −3.94	+0.19 (−0.14, 0.52; I^2^ = 52%)	4	10.97	−2.47 vs. −2.64	**+0.18 (0.01, 0.35; I**^**2**^ **= 0%)**
Mirabegron 50 mg vs. tolterodine ER 4 mg	5	2.50	−1.28 vs. −1.16	−0.12 (−0.26, 0.03; I^2^ = 0%)	4	1.01 (0.92; 1.11; I^2^ = 0%)	5	5.33	−1.90 vs. −1.88	−0.01 (−0.19, 0.17; I^2^ = 0%)	6	11.43	−1.70 vs. −1.52	−0.18 (−0.43, 0.06; I^2^ = 60%)
Fesoterodine 8 mg vs. tolterodine ER 4 mg	3	NR	−1.90 vs. −1.69	**−0.18 (−0.29, −0.07; I**^**2**^ **= 10%)**	2	**1.10 (1.04, 1.16; I**^**2**^ **= 0%)**	3	9.87	−3.68 vs. −3.14	**−0.40 (−0.69, −0.12; I**^**2**^ **= 0%)**	3	NR	−2.35 vs. −2.14	**−0.22 (−0.43, −0.01; I**^**2**^ **= 0%)**
Solifenacin 5 mg vs. tolterodine 4 mg	4	2.71	−1.56 vs. −1.25	**−0.36 (−0.58, −0.13; I**^**2**^ **= 30%)**	0	NR	4	5.53	−2.77 vs. −2.26	**−0.49 (−0.79, −0.20; I**^**2**^ **= 0%)**	4	NR	−2.35 vs. −2.14	−0.20 (−0.45, 0.05; I^2^ = 0%)
Solifenacin 5 mg vs. oxybutynin IR 15 mg	0	NR	NR	NR	0	NR	1	6.45	−2.65 vs. −3.70	+1.05 (−0.55, 2.65; I^2^ = NA)	1	NR	−2.3 vs. −3.1	+0.80 (−0.43, 2.03; I^2^ = NA)
Tolterodine vs. oxybutynin	8	5.06	−3.00 vs. −3.26	+0.01 (−0.25, 0.28; I^2^ = 57%)	1	**0.73 (0.55, 0.97; I**^**2**^ **= NA)**	0	NR	NR	NR	8	NR	−2.61 vs. −2.72	−0.16 (−0.49, 0.18; I^2^ = 30%)

Note: bold indicates statistically significant

Abbreviations: CI, confidence interval; ER, extended release; h, hour; IR, immediate release; MD, mean difference; NA, not applicable; NR, not reported; RR, risk ratio

^a^Mean at baseline calculated for studies with available data

**Table 3:** Pooled mean differences of subjective patient-reported outcomes in short-duration trial (8 to 12 weeks) of pharmacotherapies for overactive bladder

	OAB-q Symptom Bother score				PPBC			
	No. of studies	Baseline (mean)^a^	Change from baseline	MD (95% CI; I^2^)	No. of studies	Baseline (mean)^a^	Change from baseline	MD (95% CI; I^2^)
Mirabegron 50 mg + solifenacin 5 mg vs. solifenacin 5 mg	4	55.37	−28.77 vs. −23.81	−**5.02(−6.21, −3.82; I**^**2**^ **= 0%)**	4	NR	−1.49 vs. −1.21	−**0.35 (−0.48, −0.23; I**^**2**^ **= 34%)**
Mirabegron 50 mg + solifenacin 5 mg vs. mirabegron 50 mg	3	55.37	−29.40 vs. −23.89	−**6.58 (−8.15, −5.00; I**^**2**^ **= 0%)**	3	NR	−1.49 vs. −1.25	−**0.30 (−0.40, −0.20; I**^**2**^ **= 0%)**
Mirabegron 50 mg vs. solifenacin 5 mg	3	58.03	−26.62 vs. −28.16	**1.60 (0.21, 3.00; I**^**2**^ **= 9%)**	4	4.79	−1.40 vs. −1.45	0.01 (−0.11, 0.14; I^2^ = 50%)
Mirabegron 50 mg vs. tolterodine ER 4 mg	3	44.4	−17.16 vs. −16.52	−0.45 (−1.75, 0.84; I^2^ = 0%)	2	NR	−1.0 vs. −1.0	−0.02 (−0.15, 0.12; I^2^ = 0%)

Note: bold indicates statistically significant

Abbreviations: CI, confidence interval; ER, extended release; IR, immediate release; MD, mean difference; NR, not reported; OAB-q, Overactive Bladder Questionnaire; PPBC, Patient Perception of Bladder Condition

^a^Mean at baseline calculated for studies with available data

Table 4 shows pooled relative risks for adverse events. Combination therapy resulted in significantly higher incidence of constipation (3.8% vs. 2.4%) Combination therapy also showed higher rates of blurred vision (0.7–1.3% vs. 0–0.5%, Table S6) and tachycardia (1.3–10% vs. 0.3–0.7%) than monotherapy. Incidence of SAEs and other adverse events of interest were low and not significantly different between drugs.

**Table 4:** Relative risks of adverse events in short-duration trials (4 to 12 weeks) of pharmacotherapies for overactive bladder

	WAE		SAE		Constipation		Dry mouth	
	No. of studies	RR (95% CI; I^2^)	No. of studies	RR (95% CI; I^2^)	No. of studies	RR (95% CI; I^2^)	No. of studies	RR (95% CI; I^2^)
Mirabegron 50 mg + solifenacin 5 mg vs. solifenacin 5 mg	3	1.20 (0.76, 2.21; I^2^ = 0%)	3	1.81 (0.94, 3.51; I^2^ = 0%)	3	**1.65 (1.04, 2.62; I**^**2**^ **= 5%)**	3	1.13 (0.86, 1.48; I^2^ = 0%)
Mirabegron 50 mg + solifenacin 5 mg vs. mirabegron 50 mg	2	0.84 (0.29, 2.42; I^2^ = 23%)	2	1.30 (0.40, 4.16; I^2^ = 29%)	2	0.88 (0.24, 3.25; I^2^ = 54%)	2	**2.25 (1.37, 3.71; I**^**2**^ **= 0%)**
Mirabegron 50 mg vs. solifenacin 5 mg	4	0.94 (0.53, 1.68; I^2^ = 14%)	3	1.34 (0.66, 2.71; I^2^ = 6%)	3	1.16 (0.72, 1.87; I^2^ = 0%)	3	**0.53 (0.38, 0.75; I**^**2**^ **= 0%)**
Mirabegron 50 mg vs. tolterodine 4 mg	5	0.91 (0.66, 1.27; I^2^ = 0%)	4	0.89 (0.50, 1.57; I^2^ = 0%)	4	0.94 (0.58, 1.53; I^2^ = 0%)	4	**0.41 (0.25, 0.67; I**^**2**^ **= 68%)**
Fesoterodine 4 mg vs. solifenacin 5 mg	1	13.22 (0.76, 229.47; I^2^ = NA)	0	NR	1	3.05 (0.33, 28.50; I^2^ = NA)	1	2.71 (0.76, 9.73; I^2^ = NA)
Fesoterodine 8 mg vs. tolterodine 4 mg	2	**1.45 (1.07, 1.98; I**^**2**^ **= 0%)**^a^	2	**2.22 (1.21, 4.07; I**^**2**^ **= 0%)**	2	**1.41 (1.03, 1.92; I**^**2**^ **= 0%)**	2	**1.92 (1.69, 2.18; I**^**2**^ **= 0%)**
Solifenacin 5 mg vs. tolterodine 4 mg	5	1.37 (0.84, 2.23; I^2^ = 0%)^a^	0	NR	5	**2.86 (1.71, 4.78; I**^**2**^ **= 0%)**	5	1.04 (0.89, 1.22; I^2^ = 74%)^a^
Solifenacin 5 mg vs. oxybutynin IR 15 mg	1	**0.45 (0.23, 0.91; I**^**2**^ **= NA)**^a^	1	6.59 (0.35, 125.20; I^2^ = NA)	1	2.12 (0.69, 6.54; I^2^ = NA)	1	**0.43 (0.30, 0.60; I**^**2**^ **= NA)**^a^
Tolterodine vs. oxybutynin	9	**0.43 (0.27, 0.59; I**^**2**^ **= 33%)**	7	1.02 (0.55, 1.91; I^2^ = 0%)	2	1.17 (0.85, 1.62; I^2^ = 0%)	11	**0.62 (0.52, 0.74; I**^**2**^ **= 72%)**^**a**^

Note: bold indicates statistically significant

Abbreviations: CI, confidence interval; ER, extended release; IR, immediate release; NR, not reported; RR, relative risk; SAE, serious adverse event; WAE, withdrawal due to adverse event

^a^Meta-analysis result from 2012 Cochrane Systematic Review [20]

### Mirabegron plus solifenacin vs. mirabegron

Three trials (*N* = 3677) compared mirabegron plus solifenacin vs. mirabegron [29, 40, 41, 58, 61, 62, 65, 68]. Two trials lasting 12 weeks were fair quality, and one trial lasting 52 weeks was rated good quality. With the exception of proportion of patients reporting no incontinence over 3 days, mirabegron 50 mg plus solifenacin 5 mg per day significantly improved all other efficacy outcomes more than mirabegron 50 mg per day at 12 weeks (Tables 2 and 3). The greatest difference was observed in number of urgency episodes, which decreased by a mean 3.47 episodes per day with combination therapy compared with a mean 2.73 episodes per day with mirabegron. All three trials reported patient-assessed symptoms using the OAB-q Symptom Bother score and the PPBC, both of which favored combination therapy (Table 3).

Incidence of dry mouth was significantly higher with combination therapy than with mirabegron alone (8% vs. 4%), but this did not lead to significantly greater participant withdrawal (3% vs. 3%, Table 4). There were no differences between combination therapy and mirabegron monotherapy in SAE and constipation (Table 4). Incidence of blurred vision was rare and similar between treatment groups (0.8% in combination therapy vs. 0.02% in mirabegron).

### Mirabegron vs. solifenacin

**Fig. 2:**
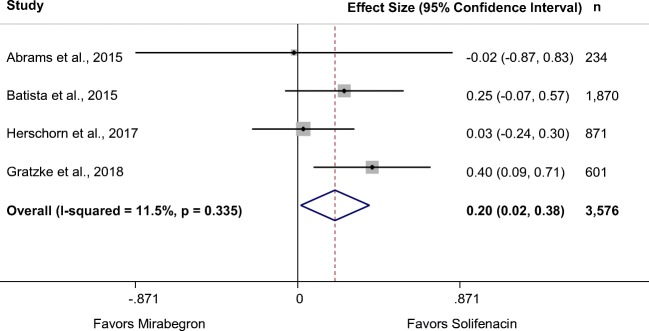
Forest plot of change from baseline number of incontinence episodes per 24 h at 12 weeks: mirabegron 50 mg vs. solifenacin 5 mg

Five RCTs in 11 records (*N* = 4279) compared mirabegron 50 mg with solifenacin 5 mg [29, 32, 40, 41, 58, 59, 61, 62, 65, 67, 68]. Two of the four larger RCTs were rated good quality, and a small, fair-quality study recruited only women. The pooled estimate based on the four larger RCTs (*N* = 3603) showed that solifenacin significantly reduced incontinence episodes more than mirabegron (Fig. 2), but there was no difference in the number of patients reporting no incontinence (51% vs. 50%, Table 2) [29, 32, 40, 41]. A significant difference was not found in reduction of urgency episodes from baseline. However, there was moderate heterogeneity (I^2^ = 52%) with three trials favoring solifenacin and one favoring mirabegron. The mean change in micturitions per day was not significantly different in individual studies, but the pooled mean difference showed solifenacin reduced micturition frequency significantly more than mirabegron (Table 2). In three RCTs, differences between the drugs in UUI (separate from overall incontinence) did not reach statistical significance.

Four trials reported patient-assessed symptoms using both the PPBC and OAB-q Symptom Bother score [29, 32, 40, 41]. There was moderate heterogeneity among trials (I^2^ = 50%) on the PPBC, though most trials and the pooled mean difference showed similar changes between treatments (Table 3). Both treatment groups achieved the MCID on the OAB-q Symptom Bother score, but solifenacin showed significantly greater improvement than mirabegron (Table 3).

Based on meta-analyses, differences in adverse events were not apparent except a small but statistically significant higher risk of dry mouth with solifenacin than with mirabegron (6.3% vs. 3.4%, Table 4). Constipation occurred at similar rates between mirabegron and solifenacin (2.2% vs. 2.1%). Pooled assessment of 12-week trials showed no difference in withdrawals due to adverse events [29, 32, 41, 67] or SAEs (Table 4) [29, 32, 41].

### Mirabegron vs. tolterodine ER

**Fig. 3:**
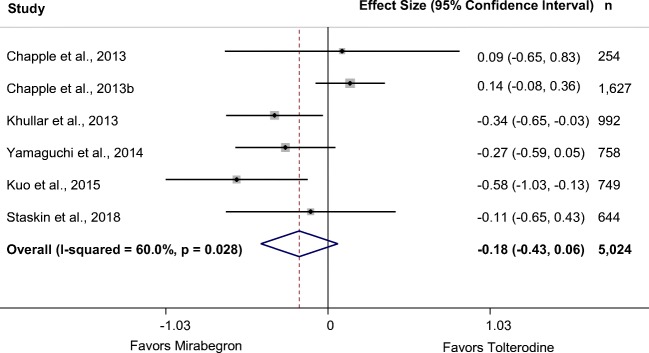
Forest plot of change from baseline number of micturitions per 24 h at 8 to 12 weeks: mirabegron 50 mg vs. tolterodine extended release (ER) 4 mg

Six RCTs in 11 publications (*N* = 4904) of mirabegron and tolterodine met the inclusion criteria for this review [30, 34, 35, 47, 48, 51, 54–57, 63, 66, 69]. All but one trial were fair quality. Meta-analyses showed no difference in any efficacy outcome between drugs. Pooling data from five trials reporting incontinence at 8 to 12 weeks found no difference between mirabegron 50 mg and tolterodine ER 4 mg (Table 2) [35, 48, 51, 66, 69]. Forty-seven percent of patients in both treatment groups reported no incontinence over 3 days at the end of treatment. In the 52-week trial, interim results at 3 months showed no significant difference between the drugs on incontinence (MD –0.01, 95% CI –0.24 to 0.22), but tolterodine ER showed greater reduction from baseline than mirabegron at study end point (MD 0.25, 95% CI 0.01 to 0.49) [35]. Change from baseline number of urgency episodes was not statistically significantly different between mirabegron and tolterodine at 8 to 12 weeks [35, 48, 51, 66, 69] and at 52 weeks [35]. Pooling six RCTs with data at 8 to 12 weeks found no significant difference between mirabegron 50 mg and tolterodine ER 4 mg in number of micturitions per day (Table 2, Fig. 3) [34, 35, 48, 51, 66, 69]. Sensitivity analysis, removing two outlier studies, did not resolve the heterogeneity. However, the difference between the drugs was very small; at 52 weeks, one study found a small, non-significant change in micturitions between mirabegron 50 mg and tolterodine ER 4 mg (MD 0.12, 95% CI –0.11 to 0.35) [35]. Three trials reported patient-assessed symptoms using the OAB-q Symptom Bother score and two also reported the PPBC, neither of which found significant differences between mirabegron 50 mg and tolterodine ER 4 mg at 8 to 12 weeks (Table 3) [35, 48, 66] or at 52 weeks [35]. All relevant treatment arms achieved the MCID on the OAB-q Symptom Bother score [35, 48, 66]. One trial also found no difference in the King’s Health Questionnaire mean change in bladder problem score [51].

Pooling results from four short-duration RCTs showed a statistically significantly higher rate of dry mouth with tolterodine 4 mg than with mirabegron 50 mg (11.7% vs. 4.6%, Table 4) [48, 51, 66, 69]. Higher incidence of dry mouth with tolterodine was also observed in the 52-week trial (8.6% vs. 2.8%) [35]. The incidence of cardiac arrhythmia was similar at 12 weeks in two trials (2.6% vs. 2.8%) [48, 51], but higher with tolterodine ER 4 mg at 12 months in a single trial (3.9% vs. 6.0%; *P* = 0.0547) [35]. However, there was no difference in the proportion of patients who withdrew because of adverse events (Table 4). Incidences of constipation, SAEs, and dizziness were similar between the drugs at both 8 to 12 weeks (Tables 4 and S6) and 52 weeks.

### Fesoterodine vs. solifenacin

One fair-quality trial (*N* = 119) compared fesoterodine 4 mg per day with solifenacin 5 mg per day [39]. The study reported only the OABSS for assessment of benefits. While fesoterodine and solifenacin each significantly improved scores from baseline at 12 weeks (−9.4 vs. −8.2) and achieved an MCID of 3 points, the difference between drugs was small and not statistically significant. Significantly more patients receiving fesoterodine compared with solifenacin withdrew because of adverse events (10.2% vs. 0.0%). Fesoterodine also resulted in higher incidence of constipation (5.1% vs. 1.7%) and dry mouth (13.6% vs. 5.0%), though differences did not reach statistical significance (*P* = 0.256 and *P* = 0.186, respectively). Other adverse events of interest were not reported.

### Fesoterodine vs. tolterodine

We did not identify any new trials that compared fesoterodine with tolterodine. The 2012 review included three 12-week trials comparing fesoterodine 8 mg per day vs. tolterodine ER 4 mg per day (*N* = 4148) [20]. Although fesoterodine led to statistically fewer incontinence and urgency episodes per day, the absolute differences were small at less than one-half episode per day for each efficacy outcome. Patients reporting no incontinence at end of treatment also favored fesoterodine (64% vs. 58%, Table 2). Fesoterodine 8 mg resulted in significantly more withdrawals due to adverse events than tolterodine ER 4 mg (4.9% vs. 3.3%, Table 4). Incidence of constipation and dry mouth was also significantly higher with fesoterodine 8 mg than with tolterodine ER 4 mg (Table 4), while there was no difference in dizziness (Table S6).

### Darifenacin vs. trospium

A small, fair-quality trial (*N* = 60) compared darifenacin 7.5 mg with trospium ER 60 mg over 4 weeks [52]. Both darifenacin and trospium ER significantly improved the OABSS composite score and achieved an MCID of 3 points, but the difference between the groups was not significant (*P* = 0.654). Similarly, both drugs significantly improved the OABSS subscales of urinary frequency (−0.80 vs. −0.47), urgency (−1.87 vs. −2.40), nocturia (−0.87 vs. −0.93), and UUI (−2.27 vs. −1.47). These differences did not reach statistical significance between drugs on these OABSS subscales. There were no SAEs or withdrawals due to adverse events. Using the McMillan & Williams Constipation Assessment Scale (0 to 16), both darifenacin and trospium significantly increased constipation compared with baseline, but there was no difference between drugs (0.93 vs. 0.60; *P* = 0.944).

### Solifenacin vs. tolterodine

Five RCTs (*N* = 2555) that compared solifenacin and tolterodine were included in the 2012 review, ranging in duration from 4 to 12 weeks, with no new studies found for this review [20]. Meta-analysis of four studies indicated that solifenacin 5 mg significantly improved incontinence and urgency episodes per day (Table 2). The difference in micturitions did not reach statistical significance (Table 2). There was no difference in withdrawals due to adverse events (3.5% for solifenacin 5 mg vs. 2.5% for tolterodine 4 mg, Table 4). There was also no difference in incidence of dry mouth, but there was high heterogeneity (I^2^ = 74%) with three trials favoring solifenacin and two favoring tolterodine. Both solifenacin 5 mg and tolterodine 4 mg resulted in blurred vision (6.2% vs. 3.8%) though the difference did not reach statistical significance. We identified one additional study [64] that compared solifenacin with tolterodine, which was not included in the 2012 systematic review, but it was rated poor quality [20].

### Solifenacin vs. oxybutynin

The 2012 review included one trial (*N* = 132) comparing solifenacin 5 mg with oxybutynin immediate release (IR) 15 mg that mainly focused on adverse effects [20], and no new evidence was found in this review. Additional data from ClinicalTrials.gov found no difference in urgency episodes per day or number of micturitions (Table 2) [53]. Fewer patients treated with solifenacin 5 mg withdrew because of adverse events than patients treated with oxybutynin IR 15 mg (Table 4). There was no difference in incidence of constipation but significantly fewer patients reported dry mouth with solifenacin than with oxybutynin (Table 4).

### Tolterodine vs. oxybutynin

The 2012 review included 14 trials (*N* = 3627) comparing tolterodine with oxybutynin [20]. We identified two subsequently published RCTs (in three publications), of which one was fair quality (*N* = 301) [46] and the other one poor quality [31, 45]. Single trials evaluated tolterodine IR 4 mg vs. oxybutynin 10 mg IR and ER, oxybutynin 9 mg IR, and transdermal oxybutynin 3.9 mg. The other trials compared IR formulations with each other: tolterodine 4 mg vs. oxybutynin 10 mg (4 RCTs), tolterodine 4 mg vs. oxybutynin 15 mg (4 RCTs), and tolterodine 1 mg vs. oxybutynin 5 mg (1 RCT). The new trial compared tolterodine IR 4 mg with oxybutynin IR 15 mg [46]. Meta-analyses for this drug comparison considered any reported daily dose.

Pooled estimates did not find statistically significant differences between drugs related to incontinence episodes per day or change in micturition frequency (Table 2), although one trial found that significantly more patients reported no incontinence with oxybutynin ER 10 mg than with tolterodine ER 4 mg (23% vs. 17%, Table 2) [70]. Only the newly identified RCT reported change in urgency episodes with no difference between tolterodine 4 mg and oxybutynin 15 mg (Table 2) [46]. Tolterodine resulted in fewer withdrawals due to adverse events compared with oxybutynin (9 RCTs, *N* = 2987) and significantly lower incidence of dry mouth (10 RCTs, *N* = 3140). Few RCTs reported on other typical anticholinergic adverse effects, with two reporting that constipation was similar between tolterodine ER/IR 4 mg and oxybutynin 10 mg (Table 4) and a single study reporting no differences between drugs related to blurred vision and dizziness (*P* = 0.393 and *P* = 0.736, respectively, Table S6).

## Discussion

Cumulatively, this systematic review update evaluated 51 head-to-head RCTs, including 20 newly identified trials. There were some statistically significant differences in efficacy between drugs but the absolute differences were small at less than half of an episode per day in key OAB outcomes (incontinence, urgency, and micturitions). The only exception was the combination of mirabegron 50 mg plus solifenacin 5 mg vs. either monotherapy on urgency episodes, where a significant difference was not found between drugs given as monotherapy, suggesting a synergistic effect. Overall, combination therapy significantly improved all efficacy outcomes assessed compared with either drug given alone. However, combination therapy also showed higher incidences of adverse events vs. monotherapy. The small potential benefit in key OAB outcomes with combination therapy should be weighed against increased risk of adverse events.

Solifenacin 5 mg showed greater improvement in incontinence and micturition frequency over mirabegron 50 mg and greater improvement in incontinence and urgency episodes over tolterodine 4 mg. There was no significant difference between mirabegron 50 mg and tolterodine 4 mg for any efficacy outcome. Patients’ assessments of symptoms as measured by the OAB-q Symptom Bother score and/or the PPBC were in accordance with these numeric measures. Pooled mean differences in the OAB-q Symptom Bother score did not reach a minimal clinically important difference, further suggesting that differences between drugs are not likely to be of clinical significance.

Although mirabegron is not an anticholinergic drug, it exhibits some adverse effects similar to anticholinergics. Pooled analyses found no difference between mirabegron 50 mg and solifenacin 5 mg or tolterodine ER 4 mg related to blurred vision, cardiac arrhythmia, constipation, or dizziness. While incidence of dry mouth was significantly lower in patients who received mirabegron, this was not reflected in the rate of withdrawal due to adverse events. At 52 weeks, the difference in incidence of dry mouth between mirabegron and solifenacin was no longer significant, though a significant difference remained between mirabegron and tolterodine. When choosing between mirabegron and solifenacin, clinicians should consider their comparable safety profile but solifenacin’s greater effectiveness on incontinence and micturition frequency.

Fesoterodine 8 mg, which is chemically related to tolterodine, showed greater improvement in incontinence, urgency episodes, and micturition frequency than tolterodine 4 mg. However, fesoterodine appeared to have a worse safety profile with significantly higher incidences of withdrawals due to adverse events, SAE, constipation, and dry mouth than tolterodine.

Based on one small trial using the OABSS, no difference was found between fesoterodine 4 mg and solifenacin 5 mg. Patients who received fesoterodine reported higher incidence of adverse events of interest, but none reached statistical significance, likely to because of the small sample size. Solifenacin 5 mg reduced one-half incontinence and urgency episodes per day more than tolterodine 4 mg, a significant difference. Overall adverse event profiles are similar between solifenacin and tolterodine, except that solifenacin led to a significantly higher incidence of constipation than tolterodine. Both fesoterodine and solifenacin are more effective than tolterodine, but clinicians should consider solifenacin for its better safety profile than fesoterodine.

From the 2012 review, oxybutynin showed comparable improvement of incontinence and micturition frequency to solifenacin 5 mg and tolterodine. The 2012 review did not evaluate patients’ assessment of symptoms, though the review found no difference between oxybutynin and tolterodine in terms of condition-specific quality of life. Significantly more patients randomized to oxybutynin withdrew because of adverse events than with solifenacin or tolterodine, likely attributed to the significantly higher incidence of dry mouth with oxybutynin.

It should be noted that the median trial duration of 3 months is longer than median time to discontinuation observed in real-world settings, which ranged from 1.0 to 3.6 months for antimuscarinics based on a systematic review by Yeowell and colleagues [71]. One-year persistence ranged from 8 to 25% for antimuscarinics and 32–38% for mirabegron [71], much lower than the 77–89% of patients who completed a 52-week trial [35, 40]. Studies included in this review showed that more patients withdrew because of adverse events than lack of efficacy. Hence, when selecting drug treatment for OAB, consideration of adverse events is as important, if not more important, than efficacy [72, 73].

Although we identified four systematic reviews/meta-analyses that have been published in recent years [74–77], our review differs in several ways. Other systematic reviews set one of the pharmacotherapies as standard therapy, comparing all other treatments to the standard, while we addressed all relevant comparisons using only direct evidence, not relying on placebo-controlled trial evidence. There have been instances where direct and indirect evidence are not consistent with each other [78]. When both direct and indirect evidence are available, the former is preferred over the latter [79, 80]. Additionally, we evaluated outcomes not included in prior reviews. We examined change in number of urgency episodes—the main complaint of patients with OAB, which distinguishes it from urinary incontinence and is a key attribute in determining patient satisfaction and persistence with treatment [73]. We also examined patient-reported assessment of symptoms to supplement quantification of symptoms. Finally, our work was not funded by the manufacturer of any of the included drugs.

Limitations of our systematic review potentially include the lack of a network meta-analysis and quality of life measures reported in some studies. These were not undertaken because of the scope, timeline, and resource limitations defined by the DERP patients who funded the initial work. Also, adverse events reporting was inconsistent among trials. Some trials reported “common anticholinergic effects,” some only reported adverse effects that affected > 2% of patients, and others reported the most common complaints reported by patients. As a result, not all harm outcomes of interest were reported by all trials, particularly blurred vision, cardiac arrhythmias, dizziness, and fall/syncope. In addition, the value and contribution of quality of life assessment in overactive bladder are unclear as Shah and Nitti pointed out [81]. Even when changes in quality of life assessments reach clinically meaningful response thresholds, persistence and patient satisfaction remain low [81]. This suggests a need to better understand what affects patient satisfaction and persistence in order to improve evaluation of available treatments.

Cumulative evidence showed small differences across all comparisons of pharmacotherapies used to treat OAB, including combination therapy (solifenacin/mirabegron) vs. monotherapy (< 0.8 episode per day). While some of these differences were statistically significant, the clinical importance is unclear, and all treatment groups reported more than one incontinence episode and 2.5 urgency episodes per day at study end. For patients with UUI at baseline, < 65% reported no incontinence over 3 days at end of treatment. Anticholinergic adverse effects remain a concern even with mirabegron. Considering that persistence with antimuscarinic drugs is already known to be low, it is unclear whether the added benefit of combining mirabegron with solifenacin outweighs the increased harm. A patient’s preference for increased efficacy vs. reduced harms, and tolerance of specific adverse events, should be considered when selecting treatment for OAB.

## Electronic supplementary material

ESM 1 (DOCX 300 kb)

## References

[1] Abrams P, Cardozo L, Fall M, Griffiths D, Rosier P, Ulmsten U, Van Kerrebroeck P, Victor A, Wein A (2002). The standardisation of terminology of lower urinary tract function: report from the standardisation sub-committee of the International Continence Society. Neurourol Urodyn.

[2] Fantl J, Newman D, Colling J, DeLancey J, Keeys C, Loughery R, McDowell B, Norton P, Ouslander J, Schnelle J (1996) Urinary Incontinence in Adults: Acute and Chronic Management. Clinical Practice Guideline No. 2, 1996 Update (AHCPR Publication No. 96–0682). Rockville, MD: US Department of Health and Human Services. Public health service, Agency for health care policy and research.

[3] Garnett S, Abrams P (2003). The natural history of the overactive bladder and detrusor overactivity. A review of the evidence regarding the long-term outcome of the overactive bladder. J Urol.

[4] Knutson T, Edlund C, Fall M, Dahlstrand C (2001). BPH with coexisting overactive bladder dysfunction—an everyday urological dilemma. Neurourol Urodyn.

[5] Gormley EA, Lightner DJ, Faraday M, Vasavada SP (2015). Diagnosis and treatment of overactive bladder (non-neurogenic) in adults: AUA/SUFU guideline amendment. J Urol.

[6] Coyne KS, Margolis MK, Kopp ZS, Kaplan SA (2012). Racial differences in the prevalence of overactive bladder in the United States from the epidemiology of LUTS (EpiLUTS) study. Urology.

[7] Coyne KS, Sexton CC, Irwin DE, Kopp ZS, Kelleher CJ, Milsom I (2008). The impact of overactive bladder, incontinence and other lower urinary tract symptoms on quality of life, work productivity, sexuality and emotional well-being in men and women: results from the EPIC study. BJU Int.

[8] Coyne KS, Wein AJ, Tubaro A, Sexton CC, Thompson CL, Kopp ZS, Aiyer LP (2009). The burden of lower urinary tract symptoms: evaluating the effect of LUTS on health-related quality of life, anxiety and depression: EpiLUTS. BJU Int.

[9] Irwin DE, Milsom I, Kopp Z, Abrams P, Cardozo L (2006). Impact of overactive bladder symptoms on employment, social interactions and emotional well-being in six European countries. BJU Int.

[10] Stewart WF, Van Rooyen JB, Cundiff GW, Abrams P, Herzog AR, Corey R, Hunt TL, Wein AJ (2003). Prevalence and burden of overactive bladder in the United States. World J Urol.

[11] Sexton CC, Coyne KS, Vats V, Kopp ZS, Irwin DE, Wagner TH (2009). Impact of overactive bladder on work productivity in the United States: results from EpiLUTS. Am J Manag Care.

[12] Abrams P, Andersson KE, Buccafusco JJ, Chapple C, de Groat WC, Fryer AD, Kay G, Laties A, Nathanson NM, Pasricha PJ, Wein AJ (2006). Muscarinic receptors: their distribution and function in body systems, and the implications for treating overactive bladder. Br J Pharmacol.

[13] Kumar V, Templeman L, Chapple CR, Chess-Williams R (2003). Recent developments in the management of detrusor overactivity. Curr Opin Urol.

[14] Rai BP, Cody JD, Alhasso A, Stewart L (2012) Anticholinergic drugs versus non-drug active therapies for non-neurogenic overactive bladder syndrome in adults. Cochrane Database of Systematic Reviews 12:CD003193. 10.1002/14651858.CD003193.pub4. 10.1002/14651858.CD003193.pub4 PMC7017858 23235594

[15] Wyndaele J-J (2011). Fesoterodine: individualised treatment of urgency urinary incontinence across patient groups. Eur Urol Suppl.

[16] Andersson KE (2017). On the site and mechanism of action of beta3-adrenoceptor agonists in the bladder. International neurourology journal.

[17] Food and Drug Administration (2012) Myrbetriq (mirabegron) Full Prescribing Information. https://www.accessdata.fda.gov/drugsatfda_docs/label/2012/202611s000lbl.pdf Accessed on April 8, 2018.

[18] Wagg AS, Foley S, Peters J, Nazir J, Kool-Houweling L, Scrine L (2017) Persistence and adherence with mirabegron vs antimuscarinics in overactive bladder: retrospective analysis of a UK general practice prescription database. Int J Clin Pract 71 (10). 10.1111/ijcp.12996. 10.1111/ijcp.12996 28906080

[19] Andersson K-E (2017). Drugs for the overactive bladder: are there differences in persistence and compliance?. Translational andrology and urology.

[20] Madhuvrata P, Cody JD, Ellis G, Herbison GP, Hay-Smith EJ (2012). Which anticholinergic drug for overactive bladder symptoms in adults. Cochrane Database Syst Rev.

[21] McDonagh MS, Jonas DE, Gartlehner G, Little A, Peterson K, Carson S, Gibson M, Helfand M (2012). Methods for the drug effectiveness review project. BMC Med Res Methodol.

[22] Methods Guide for Effectiveness and Comparative Effectiveness Reviews (January 2014). Agency for Healthcare Research and Quality, Rockville, MD. 21433403

[23] Coyne KS, Matza LS, Kopp Z, Abrams P (2006). The validation of the patient perception of bladder condition (PPBC): a single-item global measure for patients with overactive bladder. Eur Urol.

[24] Coyne KS, Matza LS, Thompson CL, Kopp ZS, Khullar V (2006). Determining the importance of change in the overactive bladder questionnaire. J Urol.

[25] Gotoh M, Homma Y, Yokoyama O, Nishizawa O (2011). Responsiveness and minimal clinically important change in overactive bladder symptom score. Urology.

[26] Sutton AJ, Duval SJ, Tweedie RL, Abrams KR, Jones DR (2000). Empirical assessment of effect of publication bias on meta-analyses. BMJ.

[27] Higgins JPT, Thompson SG (2002). Quantifying heterogeneity in a meta-analysis. Stat Med.

[28] Higgins JP, Thompson SG, Deeks JJ, Altman DG (2003). Measuring inconsistency in meta-analyses. BMJ.

[29] Abrams P, Kelleher C, Staskin D, Rechberger T, Kay R, Martina R, et al. Combination treatment with mirabegron and solifenacin in patients with overactive bladder: efficacy and safety results from a randomised, double-blind, dose-ranging, phase 2 study (Symphony). Eur Urol 67. 2015;(3):577–88. 10.1016/j.eururo.2014.02.012. 10.1016/j.eururo.2014.02.012 24612659

[30] Astellas (2010) A randomized, double-blind, parallel group, placebo and active controlled, multicenter dose ranging study with the beta-3 agonist YM178 in patients with symptomatic overactive bladder (DRAGON).1–8.

[31] Aziminekoo E, Ghanbari Z, Hashemi S, Nemati M, Haghollahi F, Shokuhi N (2014). Oxybutynin and tolterodine in a trial for treatment of overactive bladder in Iranian women. Journal of Family and Reproductive Health.

[32] Batista JE, Kolbl H, Herschorn S, Rechberger T, Cambronero J, Halaska M, Coppell A, Kaper M, Huang M, Siddiqui E (2015). The efficacy and safety of mirabegron compared with solifenacin in overactive bladder patients dissatisfied with previous antimuscarinic treatment due to lack of efficacy: results of a noninferiority, randomized, phase IIIb trial. Ther Adv Urol.

[33] But I, Goldstajn MS, Oreskovic S (2012). Comparison of two selective muscarinic receptor antagonists (solifenacin and darifenacin) in women with overactive bladder—the SOLIDAR study. Coll Antropol.

[34] Chapple CR, Dvorak V, Radziszewski P, Van Kerrebroeck P, Wyndaele JJ, Bosman B, Boerrigter P, Drogendijk T, Ridder A, Van Der Putten-Slob I, Yamaguchi O, Dragon Investigator G (2013). A phase II dose-ranging study of mirabegron in patients with overactive bladder. Int Urogynecol J Pelvic Floor Dysfunct.

[35] Chapple CR, Kaplan SA, Mitcheson D, Klecka J, Cummings J, Drogendijk T, Dorrepaal C, Martin N (2013). Randomized double-blind, active-controlled phase 3 study to assess 12-month safety and efficacy of mirabegron, a beta(3)-adrenoceptor agonist, in overactive bladder. Eur Urol.

[36] Dede H, Dolen I, Dede FS, Sivaslioglu AA (2013). What is the success of drug treatment in urge urinary incontinence? What should be measured?. Arch Gynecol Obstet.

[37] Drake MJ, Chapple C, Esen AA, Athanasiou S, Cambronero J, Mitcheson D, Herschorn S, Saleem T, Huang M, Siddiqui E, Stolzel M, Herholdt C, MacDiarmid S, investigators Bs (2016) Efficacy and safety of mirabegron add-on therapy to solifenacin in incontinent overactive bladder patients with an inadequate response to initial 4-week solifenacin monotherapy: a randomised double-blind multicentre phase 3B study (BESIDE). Eur Urol 70 (1):136–145. 10.1016/j.eururo.2016.02.030 26965560

[38] Drake MJ, MacDiarmid S, Chapple CR, Esen A, Athanasiou S, Cambronero Santos J, Mitcheson D, Herschorn S, Siddiqui E, Huang M, Stoelzel M (2017) Cardiovascular safety in refractory incontinent patients with overactive bladder receiving add-on mirabegron therapy to solifenacin (BESIDE). Int J Clin Pract 71 (5). 10.1111/ijcp.12944. 10.1111/ijcp.12944 PMC5485167 28419650

[39] Ercan O, Kostu B, Bakacak M, Aytac-Tohma Y, Coskun B, Avci F, et al. Comparison of solifenacin and fesoterodine in treatment of overactive bladder. Saudi Med J. 2015;36(10):1181–5. 10.15537/smj.2015.10.12016. 10.15537/smj.2015.10.12016 PMC4621723 26446328

[40] Gratzke C, van Maanen R, Chapple C, Abrams P, Herschorn S, Robinson D, Ridder A, Stoelzel M, Paireddy A, Yoon SJ, Al-Shukri S, Rechberger T, Mueller ER. Long-term safety and efficacy of mirabegron and solifenacin in combination compared with monotherapy in patients with overactive bladder: a randomised, multicentre phase 3 study (SYNERGY II). Eur Urol. 2018;74(4):501–509. 10.1016/j.eururo.2018.05.005. 10.1016/j.eururo.2018.05.005 29866467

[41] Herschorn S, Chapple CR, Abrams P, Arlandis S, Mitcheson D, Lee KS, Ridder A, Stoelzel M, Paireddy A, van Maanen R, Robinson D (2017). Efficacy and safety of combinations of mirabegron and solifenacin compared with monotherapy and placebo in patients with overactive bladder (SYNERGY study). BJU Int.

[42] Herschorn S, Pommerville P, Stothers L, Egerdie B, Gajewski J, Carlson K, Radomski S, Drutz H, Schulz J, Barkin J, Hirshberg E, Corcos J (2011). Tolerability of solifenacin and oxybutynin immediate release in older (> 65 years) and younger (< 65 years) patients with overactive bladder: sub-analysis from a Canadian, randomized, double-blind study. Curr Med Res Opin.

[43] Herschorn S, Staskin D, Tu LM, Fialkov J, Walsh T, Gooch K, Schermer CR (2018). Patient-reported outcomes in patients with overactive bladder treated with mirabegron and tolterodine in a prospective, double-blind, randomized, two-period crossover, multicenter study (PREFER). Health Qual Life Outcomes.

[44] Hsiao SM, Chang TC, Wu WY, Chen CH, Yu HJ, Lin HH (2011). Comparisons of urodynamic effects, therapeutic efficacy and safety of solifenacin versus tolterodine for female overactive bladder syndrome. J Obstet Gynaecol Res.

[45] Jafarabadi M, Ghanbari Z, Hashemi S, Nemati M, Haghollahi F, Azimi Nekoo E (2015). Prominent complaint: a guide to medical therapy of overactive bladder syndrome in older women. Acta Med Iran.

[46] Jafarabadi M, Jafarabadi L, Shariat M, Rabie Salehi G, Haghollahi F, Rashidi BH (2015). Considering the prominent complaint as a guide in medical therapy for overactive bladder syndrome in women over 45 years. J Obstet Gynaecol Res.

[47] Khullar V, Amarenco G, Angulo JC, Blauwet MB, Nazir J, Odeyemi IA, Hakimi Z. Patient-reported outcomes with the beta(3)-adrenoceptor agonist mirabegron in a phase III trial in patients with overactive bladder. Neurourol Urodyn. 2016;35(8):987–994 10.1002/nau.22844 26288118

[48] Khullar V, Amarenco G, Angulo JC, Cambronero J, Hoye K, Milsom I, Radziszewski P, Rechberger T, Boerrigter P, Drogendijk T, Wooning M, Chapple C (2013). Efficacy and tolerability of mirabegron, a beta(3)-adrenoceptor agonist, in patients with overactive bladder: results from a randomised European-Australian phase 3 trial. Eur Urol.

[49] Kinjo M, Sekiguchi Y, Yoshimura Y, Nutahara K. Long-term persistence with mirabegron versus solifenacin in women with overactive bladder: prospective, randomized trial. Low Urin Tract Symptoms. 2016;10(2):148–152. 10.1111/luts.12151. 10.1111/luts.12151 27911988

[50] Kosilov K, Loparev S, Ivanovskaya M, Kosilova L (2015). A randomized, controlled trial of effectiveness and safety of management of OAB symptoms in elderly men and women with standard-dosed combination of solifenacin and mirabegron. Arch Gerontol Geriatr.

[51] Kuo HC, Lee KS, Na Y, Sood R, Nakaji S, Kubota Y, Kuroishi K (2015) Results of a randomized, double-blind, parallel-group, placebo- and active-controlled, multicenter study of mirabegron, a beta3-adrenoceptor agonist, in patients with overactive bladder in Asia. Neurourol Urodyn, vol 34. 10.1002/nau.22645. 10.1002/nau.22645 25130281

[52] Manjunatha R, Pundarikaksha HP, Hanumantharaju BK, Anusha SJ (2015). A prospective, comparative study of the occurrence and severity of constipation with darifenacin and trospium in overactive bladder. J Clin Diagn Res.

[53] NCT00431041 (2010) Study to compare the safety and efficacy of solifenacin with oxybutynin for the treatment of overactive bladder (VECTOR). ClinicalTrials.gov.

[54] NCT00688688 (2013) Study to test the long-term safety and efficacy of the beta-3 agonist mirabegron (YM178) in patients with symptoms of overactive bladder (TAURUS). ClinicalTrials.gov.

[55] NCT00689104 (2013) Study to assess the efficacy and safety of the beta-3 agonist mirabegron (YM178) in patients with symptoms of overactive bladder (SCORPIO). ClinicalTrials.gov.

[56] NCT00966004 (2010) A study to evaluate safety and efficacy of YM178 in patients with overactive bladder. ClinicalTrials.gov

[57] NCT01043666 (2011) A study of YM178 in subjects with symptoms of overactive bladder. ClinicalTrials.gov

[58] NCT01340027 (2015) A study to evaluate the efficacy, safety and tolerability of mirabegron and solifenacin Succinate Alone and in combination for the treatment of overactive bladder (Symphony). ClinicalTrials.gov.

[59] NCT01638000 (2015) A study to evaluate the efficacy and safety of mirabegron compared to solifenacin in patients with overactive bladder who were previously treated with another medicine but were not satisfied with that treatment (BEYOND). ClinicalTrials.gov.

[60] NCT01908829 (2016) A trial comparing combination treatment (solifenacin plus mirabegron) with one treatment alone (Solifenacin) (BESIDE). ClinicalTrials.gov.

[61] NCT01972841 (2017) This was a multinational study comparing the efficacy and safety of two medicines, solifenacin succinate and mirabegron taken together, or separately, or a mock treatment (placebo) in subjects with symptoms of overactive bladder (SYNERGY). ClinicalTrials.gov.

[62] NCT02045862 (2018) A multinational study comparing the long-term efficacy and safety of two medicines, solifenacin succinate and mirabegron taken together, or separately, in subjects with symptoms of overactive bladder (SYNERGY II). ClinicalTrials.gov.

[63] NCT02138747 (2018) A study to evaluate tolerability and participants’ preference between mirabegron and tolterodine extended release (ER) in participants with overactive bladder (OAB) (PREFER). ClinicalTrials.gov.

[64] Rana M, Mobusher I (2016). Comparison of side effects of tolterodine and solifenacinsucinate in patients with urinary incontinence. Pakistan Journal of Medical and Health Sciences.

[65] Robinson D, Kelleher C, Staskin D, Mueller ER, Falconer C, Wang J, Ridder A, Stoelzel M, Paireddy A, van Maanen R, Hakimi Z, Herschorn S Patient-reported outcomes from SYNERGY, a randomized, double-blind, multicenter study evaluating combinations of mirabegron and solifenacin compared with monotherapy and placebo in OAB patients. Neurourol Urodyn. 2017;37(1):394–406. 10.1002/nau.23315. 10.1002/nau.23315 28704584

[66] Staskin D, Herschorn S, Fialkov J, Tu LM, Walsh T, Schermer CR (2018). A prospective, double-blind, randomized, two-period crossover, multicenter study to evaluate tolerability and patient preference between mirabegron and tolterodine in patients with overactive bladder (PREFER study). Int Urogynecol J Pelvic Floor Dysfunct.

[67] Vecchioli Scaldazza C, Morosetti C (2016). Comparison of therapeutic efficacy and urodynamic findings of solifenacin succinate versus mirabegron in women with overactive bladder syndrome: results of a randomized controlled study. Urol Int.

[68] White WB, Chapple C, Gratzke C, Herschorn S, Robinson D, Frankel J, Ridder A, Stoelzel M, Paireddy A, van Maanen R, Weber MA. Cardiovascular safety of the beta(3)-adrenoceptor agonist mirabegron and the antimuscarinic agent solifenacin in the SYNERGY trial. J Clin Pharmacology. 2018;58(8):1084–1091. 10.1002/jcph.1107. 10.1002/jcph.1107 29645285

[69] Yamaguchi O, Marui E, Kakizaki H, Homma Y, Igawa Y, Takeda M, Nishizawa O, Gotoh M, Yoshida M, Yokoyama O, Seki N, Ikeda Y, Ohkawa S (2014). Phase III, randomised, double-blind, placebo-controlled study of the beta3-adrenoceptor agonist mirabegron, 50mg once daily, in Japanese patients with overactive bladder. BJU Int.

[70] Diokno AC, Appell RA, Sand PK, Dmochowski RR, Gburek BM, Klimberg IW, Kell SH (2003). Prospective, randomized, double-blind study of the efficacy and tolerability of the extended-release formulations of oxybutynin and tolterodine for overactive bladder: results of the OPERA trial. Mayo Clin Proc.

[71] Yeowell G, Smith P, Nazir J, Hakimi Z, Siddiqui E, Fatoye F (2018). Real-world persistence and adherence to oral antimuscarinics and mirabegron in patients with overactive bladder (OAB): a systematic literature review. BMJ Open.

[72] Akino H, Namiki M, Suzuki K, Fuse H, Kitagawa Y, Miyazawa K, Fujiuchi Y, Yokoyama O (2014). Factors influencing patient satisfaction with antimuscarinic treatment of overactive bladder syndrome: results of a real-life clinical study. Int J Urol.

[73] Heisen M, Baeten SA, Verheggen BG, Stoelzel M, Hakimi Z, Ridder A, van Maanen R, Stolk EA (2016). Patient and physician preferences for oral pharmacotherapy for overactive bladder: two discrete choice experiments. Curr Med Res Opin.

[74] Maman K, Aballea S, Nazir J, Desroziers K, Neine ME, Siddiqui E, Odeyemi I, Hakimi Z (2014). Comparative efficacy and safety of medical treatments for the management of overactive bladder: a systematic literature review and mixed treatment comparison. Eur Urol.

[75] Drake MJ, Nitti VW, Ginsberg DA, Brucker BM, Hepp Z, McCool R, Glanville JM, Fleetwood K, James D, Chapple CR (2017). Comparative assessment of the efficacy of onabotulinumtoxinA and oral therapies (anticholinergics and mirabegron) for overactive bladder: a systematic review and network meta-analysis. BJU Int.

[76] Kelleher C, Hakimi Z, Zur R, Siddiqui E, Maman K, Aballea S, Nazir J, Chapple C (2018). Efficacy and tolerability of Mirabegron compared with Antimuscarinic monotherapy or combination therapies for overactive bladder: a systematic review and network meta-analysis. Eur Urol.

[77] Obloza A, Kirby J, Yates D, Toozs-Hobson P (2017). Indirect treatment comparison (ITC) of medical therapies for an overactive bladder. Neurourol Urodyn.

[78] Chou R, Carson S, Chan BK (2009). Gabapentin versus tricyclic antidepressants for diabetic neuropathy and post-herpetic neuralgia: discrepancies between direct and indirect meta-analyses of randomized controlled trials. J Gen Intern Med.

[79] National Institute for Health and Care Excellence (2012). The guidelines manual.

[80] Higgins J, Green S, (editors) (2011) Cochrane Handbook for Systematic Reviews of Interventions Version 5.1.0 [updated March 2011]. The Cochrane Collaboration:Section 16.16.12. Available from www.handbook.cochrane.org

[81] Shah S, Nitti VW (2009). Defining efficacy in the treatment of overactive bladder syndrome. Reviews in urology.

