# Supplementary material for: Updating the evidence on drugs to treat overactive bladder: a systematic review
Source: Int Urogynecol J. 2019 Jul 25;30(10):1603–17. doi: 10.1007/s00192-019-04022-8 (PMC6795617; doi:10.1007/s00192-019-04022-8)
Supplement: Supplementary file 1 — (DOCX 300 kb) [file 192_2019_4022_MOESM1_ESM.docx]

**Table S1. Literature Search Strategies**

| MEDLINE | 1. mirabegron.mp. |
| --- | --- |
|  | 1. Myrbetriq.mp. |
|  | 1. fesoterodine.mp. |
|  | 1. Toviaz.mp. |
|  | 1. darifenacin.mp. |
|  | 1. Enablex.mp. |
|  | 1. solifenacin.mp. |
|  | 1. Vesicare.mp. |
|  | 1. trospium.mp. |
|  | 1. oxybutynin.mp. |
|  | 1. Ditropan.mp. |
|  | 1. Oxytrol.mp. |
|  | 1. Gelnique.mp. |
|  | 1. tolterodine.mp. |
|  | 1. Detrol.mp. |
|  | 1. flavoxate.mp. |
|  | 1. 1 or 2 or 3 or 4 or 5 or 6 or 7 or 8 or 9 or 10 or 11 or 12 or 13 or 14 or 15 or 16 |
|  | 1. Limit 17 to yr=”2011 –Current” |
|  | 1. limit 18 to english language (82) |
|  | 1. limit 19 to clinical trial, all (31) |
|  | 1. limit 20 to animals |
|  | 1. 20 not 21 |
| MEDLINE In-Process | 1. mirabegron.mp. |
|  | 1. Myrbetriq.mp. |
|  | 1. fesoterodine.mp. |
|  | 1. Toviaz.mp. |
|  | 1. darifenacin.mp. |
|  | 1. Enablex.mp. |
|  | 1. solifenacin.mp. |
|  | 1. Vesicare.mp. |
|  | 1. trospium.mp. |
|  | 1. oxybutynin.mp. |
|  | 1. Ditropan.mp. |
|  | 1. Oxytrol.mp. |
|  | 1. Gelnique.mp. |
|  | 1. tolterodine.mp. |
|  | 1. Detrol.mp. |
|  | 1. flavoxate.mp. |
|  | 1. 1 or 2 or 3 or 4 or 5 or 6 or 7 or 8 or 9 or 10 or 11 or 12 or 13 or 14 or 15 or 16 |
|  | 1. (2017$ or 2018$).ed. |
|  | 1. 17 and 18 |
|  | 1. Limit 19 to english language |
| Cochrane Central Register of Controlled Trials | 1. mirabegron.mp. |
|  | 1. Myrbetriq.mp. |
|  | 1. fesoterodine.mp. |
|  | 1. Toviaz.mp. |
|  | 1. darifenacin.mp. |
|  | 1. Enablex.mp. |
|  | 1. solifenacin.mp. |
|  | 1. Vesicare.mp. |
|  | 1. trospium.mp. |
|  | 1. oxybutynin.mp. |
|  | 1. Ditropan.mp. |
|  | 1. Oxytrol.mp. |
|  | 1. Gelnique.mp. |
|  | 1. tolterodine.mp. |
|  | 1. Detrol.mp. |
|  | 1. flavoxate.mp. |
|  | 1. 1 or 2 or 3 or 4 or 5 or 6 or 7 or 8 or 9 or 10 or 11 or 12 or 13 or 14 or 15 or 16 |
|  | 1. Limit 17 to yr=”2011 –Current” |
|  | 1. Limit 18 to english language |
| Cochrane Database of Systematic Reviews | 1. mirabegron.mp. |
|  | 1. Myrbetriq.mp. |
|  | 1. fesoterodine.mp. |
|  | 1. Toviaz.mp. |
|  | 1. darifenacin.mp. |
|  | 1. Enablex.mp. |
|  | 1. solifenacin.mp. |
|  | 1. Vesicare.mp. |
|  | 1. trospium.mp. |
|  | 1. oxybutynin.mp. |
|  | 1. Ditropan.mp. |
|  | 1. Oxytrol.mp. |
|  | 1. Gelnique.mp. |
|  | 1. tolterodine.mp. |
|  | 1. Detrol.mp. |
|  | 1. flavoxate.mp. |
|  | 1. 1 or 2 or 3 or 4 or 5 or 6 or 7 or 8 or 9 or 10 or 11 or 12 or 13 or 14 or 15 or 16 |
|  | 1. Limit 17 to last 5 years |

**Table S2. List of Included Studies**

| Primary publications of randomized controlled trials | Abrams P, Kelleher C, Staskin D, Rechberger T, Kay R, Martina R, et al. Combination treatment with mirabegron and solifenacin in patients with overactive bladder: efficacy and safety results from a randomised, double-blind, dose-ranging, phase 2 study (Symphony). Eur Urol. 2015;67(3):577-88. |
| --- | --- |
|  | Aziminekoo E, Ghanbari Z, Hashemi S, Nemati M, Haghollahi F, Shokuhi N. Oxybutynin and tolterodine in a trial for treatment of overactive bladder in Iranian women. Journal of Family and Reproductive Health. 2014;8(2):73-6. |
|  | Batista JE, Kolbl H, Herschorn S, Rechberger T, Cambronero J, Halaska M, et al. The efficacy and safety of mirabegron compared with solifenacin in overactive bladder patients dissatisfied with previous antimuscarinic treatment due to lack of efficacy: Results of a noninferiority, randomized, phase IIIb trial. Therapeutic advances in urology. 2015;7(4):167-79. |
|  | Chapple CR, Dvorak V, Radziszewski P, et al. A phase II dose-ranging study of mirabegron in patients with overactive bladder. Int Urogynecol J Pelvic Floor Dysfunct. 2013;24(9):1447-1458. |
|  | Chapple CR, Kaplan SA, Mitcheson D, Klecka J, Cummings J, Drogendijk T, et al. Randomized double-blind, active-controlled phase 3 study to assess 12-month safety and efficacy of mirabegron, a beta(3)-adrenoceptor agonist, in overactive bladder. Eur Urol. 2013;63(2):296-305. |
|  | Dede H, Dolen I, Dede FS, Sivaslioglu AA. What is the success of drug treatment in urge urinary incontinence? What should be measured? Arch Gynecol Obstet. 2013;287(3):511-8. |
|  | Drake MJ, Chapple C, Esen AA, et al. Efficacy and safety of mirabegron add-on therapy to solifenacin in incontinent overactive bladder patients with an inadequate response to initial 4-week solifenacin monotherapy: a randomised double-blind multicentre phase 3B study (BESIDE). Eur Urol. 2016;70(1):136-145. |
|  | Ercan O, Kostu B, Bakacak M, Aytac-Tohma Y, Coskun B, Avci F, et al. Comparison of solifenacin and fesoterodine in treatment of overactive bladder. Saudi Med J. 2015;36(10):1181-5. |
|  | Gratzke C, van Maanen R, Chapple C, Abrams P, Herschorn S, Robinson D, et al. Long-term Safety and Efficacy of Mirabegron and Solifenacin in Combination Compared with Monotherapy in Patients with Overactive Bladder: a Randomised, Multicentre Phase 3 Study (SYNERGY II). Eur Urol. 2018(pagination). |
|  | Herschorn S, Chapple CR, Abrams P, Arlandis S, Mitcheson D, Lee KS, et al. Efficacy and safety of combinations of mirabegron and solifenacin compared with monotherapy and placebo in patients with overactive bladder (SYNERGY study). BJU Int. 2017;120(4):562-75. |
|  | Jafarabadi M, Jafarabadi L, Shariat M, Rabie Salehi G, Haghollahi F, Rashidi BH. Considering the prominent complaint as a guide in medical therapy for overactive bladder syndrome in women over 45 years. J Obstet Gynaecol Res. 2015;41(1):120-6. |
|  | Khullar V, Amarenco G, Angulo JC, Cambronero J, Hoye K, Milsom I, et al. Efficacy and tolerability of mirabegron, a beta(3)-adrenoceptor agonist, in patients with overactive bladder: results from a randomised European-Australian phase 3 trial. Eur Urol. 2013;63(2):283-95. |
|  | Kinjo M, Sekiguchi Y, Yoshimura Y, Nutahara K. Long-term Persistence with Mirabegron versus Solifenacin in Women with Overactive Bladder: prospective, Randomized Trial. LUTS: lower urinary tract symptoms. 2016(pagination). |
|  | Kosilov K, Loparev S, Ivanovskaya M, Kosilova L. A randomized, controlled trial of effectiveness and safety of management of OAB symptoms in elderly men and women with standard-dosed combination of solifenacin and mirabegron. Arch Gerontol Geriatr. 2015;61(2):212-6. |
|  | Kuo HC, Lee KS, Na Y, Sood R, Nakaji S, Kubota Y, et al. Results of a randomized, double-blind, parallel-group, placebo- and active-controlled, multicenter study of mirabegron, a beta3-adrenoceptor agonist, in patients with overactive bladder in Asia. Neurourol Urodyn2015. p. 685-92. |
|  | Manjunatha R, Pundarikaksha HP, Hanumantharaju BK, Anusha SJ. A prospective, comparative study of the occurrence and severity of constipation with darifenacin and trospium in overactive bladder. Journal of Clinical and Diagnostic Research. 2015;9(3):FC05-FC9. |
|  | Rana M, Mobusher I. Comparison of side effects of tolterodine and solifenacinsucinate in patients with urinary incontinence. Pakistan Journal of Medical and Health Sciences. 2016;10(1):176-80. |
|  | Staskin D, Herschorn S, Fialkov J, Tu LM, Walsh T, Schermer CR. A prospective, double-blind, randomized, two-period crossover, multicenter study to evaluate tolerability and patient preference between mirabegron and tolterodine in patients with overactive bladder (PREFER study). Int Urogynecol J Pelvic Floor Dysfunct. 2018;29(2):273-83. |
|  | Vecchioli Scaldazza C, Morosetti C. Comparison of Therapeutic Efficacy and Urodynamic Findings of Solifenacin Succinate versus Mirabegron in Women with Overactive Bladder Syndrome: Results of a Randomized Controlled Study. Urol Int. 2016;97(3):325-9. |
|  | Yamaguchi O, Marui E, Kakizaki H, Homma Y, Igawa Y, Takeda M, et al. Phase III, randomised, double-blind, placebo-controlled study of the beta3-adrenoceptor agonist mirabegron, 50mg once daily, in Japanese patients with overactive bladder. BJU Int. 2014;113(6):951-60. |
| Secondary publications randomized controlled trials | Astellas. A randomized, double-blind, parallel group, placebo and active controlled, multicenter dose ranging study with the beta-3 agonist YM178 in patients with symptomatic overactive bladder (DRAGON). 2010:1-8. |
|  | Drake MJ, MacDiarmid S, Chapple CR, et al. Cardiovascular safety in refractory incontinent patients with overactive bladder receiving add-on mirabegron therapy to solifenacin (BESIDE). Int J Clin Pract. 2017;71(5). |
|  | Herschorn S, Staskin D, Tu LM, Fialkov J, Walsh T, Gooch K, et al. Patient-reported outcomes in patients with overactive bladder treated with mirabegron and tolterodine in a prospective, double-blind, randomized, two-period crossover, multicenter study (PREFER). Health Qual Life Outcomes. 2018;16(1):69. |
|  | Jafarabadi M, Ghanbari Z, Hashemi S, Nemati M, Haghollahi F, Azimi Nekoo E. Prominent complaint: a guide to medical therapy of overactive bladder syndrome in older women. Acta Med Iran. 2015;53(2):125-8. |
|  | NCT00431041. Study to Compare the Safety and Efficacy of Solifenacin With Oxybutynin for the Treatment of Overactive Bladder (VECTOR). In. ClinicalTrials.gov. 2010. |
|  | NCT00688688. Study to Test the Long Term Safety and Efficacy of the Beta-3 Agonist Mirabegron (YM178) in Patients With Symptoms of Overactive Bladder (TAURUS). In. ClinicalTrials.gov2013. |
|  | NCT00689104. Study to Assess the Efficacy and Safety of the Beta-3 Agonist Mirabegron (YM178) in Patients With Symptoms of Overactive Bladder (SCORPIO). In. ClinicalTrials.gov2013. |
|  | NCT00966004. A Study to Evaluate Safety and Efficacy of YM178 in Patients With Overactive Bladder. In:2010. |
|  | NCT01043666. A Study of YM178 in Subjects With Symptoms of Overactive Bladder. In:2011. |
|  | NCT01340027. A Study to Evaluate the Efficacy, Safety and Tolerability of Mirabegron and Solifenacin Succinate Alone and in Combination for the Treatment of Overactive Bladder (Symphony). In. ClinicalTrials.gov2015. |
|  | NCT01638000. A Study to Evaluate the Efficacy and Safety of Mirabegron Compared to Solifenacin in Patients With Overactive Bladder Who Were Previously Treated With Another Medicine But Were Not Satisfied With That Treatment (BEYOND). In. ClinicalTrials.gov2015. |
|  | NCT01908829. A Trial Comparing Combination Treatment (Solifenacin Plus Mirabegron) With One Treatment Alone (Solifenacin) (BESIDE). In. ClinicalTrials.gov2016. |
|  | NCT01972841. This Was a Multinational Study Comparing the Efficacy and Safety of Two Medicines , Solifenacin Succinate and Mirabegron Taken Together, or Separately, or a Mock Treatment (Placebo) in Subjects With Symptoms of Overactive Bladder (SYNERGY). In. ClinicalTrials.gov2017. |
|  | NCT02045862. A Multinational Study Comparing the Long-term Efficacy and Safety of Two Medicines, Solifenacin Succinate and Mirabegron Taken Together, or Separately, in Subjects With Symptoms of Overactive Bladder (SYNERGY II). In. ClinicalTrials.gov2018. |
|  | NCT02138747. A Study to Evaluate Tolerability and Participants Preference Between Mirabegron and Tolterodine Extended Release (ER) in Participants With Overactive Bladder (OAB) (PREFER). In. ClinicalTrials.gov2018. |
|  | Khullar V, Amarenco G, Angulo JC, Blauwet MB, Nazir J, Odeyemi IA, et al. Patient-reported outcomes with the beta<sub>3</sub> -adrenoceptor agonist mirabegron in a phase III trial in patients with overactive bladder. Neurourol Urodyn. 2016. p. 987-94. |
|  | Robinson D, Kelleher C, Staskin D, Mueller ER, Falconer C, Wang J, et al. Patient-reported outcomes from SYNERGY, a randomized, double-blind, multicenter study evaluating combinations of mirabegron and solifenacin compared with monotherapy and placebo in OAB patients. Neurourology and urodynamics. 2017(pagination). |
|  | White WB, Chapple C, Gratzke C, Herschorn S, Robinson D, Frankel J, et al. Cardiovascular Safety of the beta<inf>3</inf>-Adrenoceptor Agonist Mirabegron and the Antimuscarinic Agent Solifenacin in the SYNERGY Trial. Journal of clinical pharmacology. 2018(pagination). |
| Secondary publications of previously included trials | But I, Goldstajn MS, Oreskovic S. Comparison of two selective muscarinic receptor antagonists (solifenacin and darifenacin) in women with overactive bladder--the SOLIDAR study. Coll Antropol. 2012;36(4):1347-53. |
|  | Herschorn S, Pommerville P, Stothers L, Egerdie B, Gajewski J, Carlson K, et al. Tolerability of solifenacin and oxybutynin immediate release in older (> 65 years) and younger (< 65 years) patients with overactive bladder: sub-analysis from a Canadian, randomized, double-blind study. Curr Med Res Opin. 2011;27(2):375-82. |
|  | Hsiao SM, Chang TC, Wu WY, Chen CH, Yu HJ, Lin HH. Comparisons of urodynamic effects, therapeutic efficacy and safety of solifenacin versus tolterodine for female overactive bladder syndrome. J Obstet Gynaecol Res. 2011;37(8):1084-91. |

**Table S3. List of Excluded Studies**

The following full-text publications were considered for inclusion but failed to meet the criteria for this review.

Exclusion codes: 1 = Foreign language; 2 = Outcome not included; 3 = Intervention not included; 4 = Population not included; 5 = Publication type not included; 6 = Study design not included; 7 = Study not obtainable; 8 = Outdated or ineligible systematic review

| Abrams P, Kelleher C, Staskin D, Kay R, Martan A, Mincik I, et al. Combination treatment with mirabegron and solifenacin in patients with overactive bladder: exploratory responder analyses of efficacy and evaluation of patient-reported outcomes from a randomized, double-blind, factorial, dose-ranging, Phase II study (SYMPHONY). World J Urol. 2017;35(5):827-38. | 4 |
| --- | --- |
| Alcantara-Montero A. Combined treatment of solifenacin and mirabegron, an alternative in patients with overactive bladder (BESIDE study). Actas Urol Esp. 2016;40(9):593-4. | 6 |
| Amarenco G, Sutory M, Fagertun H, Wright M, Compion G, De Ridder D. Solifenacin is effective and well tolerated in patients with neurogenic detrusor overactivity: Preliminary results from the SONIC urodynamic study. European Urology, Supplements. 2017;11(1):e467-ea. | 4 |
| Amarenco G, Sutory M, Zachoval R, Agarwal M, Del Popolo G, Tretter R, et al. Solifenacin is effective and well tolerated in patients with neurogenic detrusor overactivity: Results from the double-blind, randomized, active- and placebo-controlled SONIC urodynamic study. Neurourol Urodyn. 2017;36(2):414-21. | 3 |
| Andersson KE. Re: Nonantimuscarinic Treatment for Overactive Bladder: A Systematic Review. Eur Urol. 2016;70(6):1077. | 5 |
| Barkin J, Diles D, Franks B, Berner T. Alpha blocker monotherapy versus combination therapy with antimuscarinics in men with persistent LUTS refractory to alpha-adrenergic treatment: patterns of persistence. Canadian Journal of Urology. 2015;22(4):7914-23. | 4 |
| Bunniran S, Davis C, Kristy R, Ng D, Schermer CR, Uribe C, et al. A prospective study of elderly initiating mirabegron versus antimuscarinics: patient reported outcomes from the Overactive Bladder Satisfaction Scales and other instruments. Neurourology and urodynamics2017. | 6 |
| Chapple C, Oelke M, Kaplan SA, Scholfield D, Arumi D, Wagg AS. Fesoterodine clinical efficacy and safety for the treatment of overactive bladder in relation to patient profiles: a systematic review. Curr Med Res Opin. 2015;31(6):1201-43. | 8 |
| Chua EM, Lapitan CM, Silangcruz MJ, Luna S, Jr., Morales LM, Jr. Beta-3 adrenergic receptor agonist for adult with overactive bladder. Cochrane Database of Systematic Reviews. 2015(3). | 5 |
| Corcos J, Angulo JC, Garely AD, Carlsson M, Gong J, Guan Z, et al. Effect of fesoterodine 4 mg on bladder diary and patient-reported outcomes during the first week of treatment in subjects with overactive bladder. Curr Med Res Opin. 2011;27(5):1059-65. | 6 |
| Cornu JN. Combination of solifenacin and mirabegron for overactive bladder management. BJU Int. 2015;116(4):498-9. | 5 |
| Dmochowski R, Duchin K, Tremblay T, Paborji M, Flugel R. TolenixTM (THVD-201), a novel combination of muscarinic agonist (tolterodine) and muscarinic agonist (pilocarpine), is efficacious in OAB with less dry mouth compared to tolterodine alone. European Urology, Supplements. 2013;12(1):e402-e3. | 3 |
| Drake M, Adil Esen A, Athanasiou S, Herholdt C, Kaper M, Saleem T, et al. Safety and efficacy of mirabegron add-on treatment to solifenacin in incontinent oab subjects with an inadequate response to initial 4-week solifenacin monotherapy. Neurourology and urodynamics. 2015;34(6). | 5 |
| Drake MJ, Chapple C, Esen AA, Athanasiou S, Cambronero J, Mitcheson D, et al. Efficacy and Safety of Mirabegron Add-on Therapy to Solifenacin in Incontinent Overactive Bladder Patients with an Inadequate Response to Initial 4-Week Solifenacin Monotherapy: A Randomised Double-blind Multicentre Phase 3B Study (BESIDE). Eur Urol. 2016;70(1):136-45. | 3 |
| Drake MJ, Nitti VW, Ginsberg DA, Brucker BM, Hepp Z, McCool R, et al. Comparative assessment of the efficacy of onabotulinumtoxinA and oral therapies (anticholinergics and mirabegron) for overactive bladder: a systematic review and network meta-analysis. BJU Int. 2017;120(5):611-22. | 8 |
| Drake MJ, Oelke M, Snijder R, Klaver M, Traudtner K, van Charldorp K, et al. Incidence of urinary retention during treatment with single tablet combinations of solifenacin+tamsulosin OCASTM for up to 1 year in adult men with both storage and voiding LUTS: A subanalysis of the NEPTUNE/NEPTUNE II randomized controlled studies. PLoS ONE [Electronic Resource]. 2017;12(2):e0170726. | 3 |
| Edwards SJ, Karner C, Trevor N, Barton S, Nherera L. Comparisons of the clinical effectiveness of treatments for the symptoms associated with ov eractive bladder (OAB). Value Health. 2013;16(7):A630. | 5 |
| Esin E, Ergen A, Cankurtaran M, Yavuz BB, Halil M, Ulger Z, et al. Influence of antimuscarinic therapy on cognitive functions and quality of life in geriatric patients treated for overactive bladder. Aging & Mental Health. 2015;19(3):217-23. | 6 |
| Fukuda T, Yamanishi T, Uchiyama T, Kamai T. Randomized, Single-Blind, Parallel Study of the Effectiveness and Safety of Solifenacin versus Propiverine in the Treatment of Overactive Bladder. LUTS: Lower Urinary Tract Symptoms. 2013;5(1):11-6. | 3 |
| Ghanbari Z, Eftekhar T, Esmaeili M, Miri E. Comparison of efficacy and side-effects of oxybutynin and tolterodine in the treatment of overactive bladder. Tehran university medical journal. 2011;69(5):302-8. | 1 |
| Gibson W, MacDiarmid S, Huang M, Siddiqui E, Stolzel M, Choudhury N, et al. Treating Overactive Bladder in Older Patients with a Combination of Mirabegron and Solifenacin: a Prespecified Analysis from the BESIDE Study. European urology focus. 2017(pagination). | 6 |
| Ginsberg D, Schneider T, Kelleher C, Van Kerrebroeck P, Swift S, Creanga D, et al. Efficacy of fesoterodine compared with extended-release tolterodine in men and women with overactive bladder. BJU Int. 2013;112(3):373-85. | 6 |
| Gittelman M, Weiss H, Seidman L. A phase 2, randomized, double-blind, efficacy and safety study of oxybutynin vaginal ring for alleviation of overactive bladder symptoms in women. The Journal of urology. 2014;191(4):1014-21. | 3 |
| Gray SL, Anderson ML, Dublin S, et al. Cumulative use of strong anticholinergics and incident dementia: A prospective cohort study. JAMA Internal Medicine. 2015;175(3):401-7. | 3 |
| Hekal IA. Drug treatment of bothersome lower urinary tract symptoms after ureteric JJ-stent insertion: a contemporary, comparative, prospective, randomised placebo-controlled study, single-centre experience. Arab journal of urology. 2016;14(4):262-8. | 4 |
| Herdman M, Nazir J, Hakimi Z, Siddiqui E, Huang M, Pavesi M, et al. Assessing preference-based outcome measures for overactive bladder: an evaluation of patient-reported outcome data from the BESIDE clinical trial. Patient. 2017. | 6 |
| Herschorn S, Kaplan SA, Sun F, Ntanios F. Do patient characteristics predict responsiveness to treatment of overactive bladder with antimuscarinic agents? Urology. 2014;83(5):1023-9. | 6 |
| Herschorn S, Kohan A, Aliotta P, McCammon K, Sriram R, Abrams S, et al. The Efficacy and Safety of OnabotulinumtoxinA or Solifenacin Compared with Placebo in Solifenacin Naive Patients with Refractory Overactive Bladder: results from a Multicenter, Randomized, Double-Blind Phase 3b Trial. J Urol. 2017(pagination). | 3 |
| Herschorn S, Kowey P, Nitti V, Milsom I, Auerbach SM, Blauwet MB, et al. Cardiovascular (CV) assessments in short- and long-term Phase 3 mirabegron trials in patients with overactive bladder (OAB). European Urology, Supplements. 2013;12(1):e739-e40. | 5 |
| Huang W, Zong H, Zhou X, Zhang Y. Efficacy and safety of imidafenacin for overactive bladder in adult: a systematic review and meta-analysis. Int Urol Nephrol. 2015;47(3):457-64. | 8 |
| Kalder M, Pantazis K, Dinas K, Albert US, Heilmaier C, Kostev K. Discontinuation of treatment using anticholinergic medications in patients with urinary incontinence. Obstetrics & Gynecology. 2014;124(4):794-800. | 2 |
| Kallner HK, Elmer C, Andersson KE, Altman D. Hormonal influence on the effect of mirabegron treatment for overactive bladder. Menopause. 2016;23(12):1303-6. | 6 |
| Kaplan SA. Re: beta3-adrenoreceptor agonist mirabegron is effective for overactive bladder that is unresponsive to antimuscarinic treatment or is related to benign prostatic hyperplasia in men. J Urol. 2014;191(5):1344-6. | 5 |
| Kazemi Rashed F, Nourizade D, Hajebrahimi S, Hasanzade K, Otoofat A. Does combination therapy with desmopressin and tolterodine improve the treatment outcomes of patients with monosymptomatic nocturnal enuresis? A randomized clinical controlled trial. ISRN urology. 2013;2013(413146). | 4 |
| Kelleher C, Aballea S, Maman K, Nazir J, Hakimi Z, Chambers C, et al. Comparative efficacy and tolerability of solifenacin 5mg versus oral antimuscarinic agents in overactive bladder (OAB): A systematic literature review (SLR) and mixed treatment comparison (MTC). Value Health. 2014;17(7):A466. | 5 |
| Kim TH, Lee SE, Lee HE, Lee KS. Safety and efficacy of fesoterodine fumarate in patients with overactive bladder: results of a post-marketing surveillance study in Korea. Curr Med Res Opin. 2016;32(8):1361-6. | 6 |
| Kosilov KV, Loparev SA, Ivanovskaya MA, Kosilova LV. Effectiveness of Solifenacin and Trospium for Managing of Severe Symptoms of Overactive Bladder in Patients With Benign Prostatic Hyperplasia. Am j. 2016;10(2):157-63. | 4 |
| Kosilov KV, Loparev SA, Ivanovskaya MA, Kosilova LV. Influence of different doses of trospium and solifenacin on manageability of OAB symptoms with different severity in elderly men and women. Journal of Clinical Urology. 2016;9(3):180-8. | 6 |
| Kosilov KV, Loparev SA, Ivanovskaya MA, Kosilova LV. Randomized controlled trial of cyclic and continuous therapy with trospium and solifenacin combination for severe overactive bladder in elderly patients with regard to patient compliance. Therapeutic advances in urology. 2014;6(6):215-23. | 6 |
| Krhut J, Gartner M, Petzel M, Sykora R, Nemec D, Tvrdik J, et al. Persistence with first line anticholinergic medication in treatment-naive overactive bladder patients. Scandinavian Journal of Urology. 2014;48(1):79-83. | 6 |
| Lee KS, Park B, Kim JH, Kim HG, Seo JT, Lee JG, et al. A randomised, double-blind, parallel design, multi-institutional, non-inferiority phase IV trial of imidafenacin versus fesoterodine for overactive bladder. Int J Clin Pract. 2013;67(12):1317-26. | 3 |
| MacDiarmid S, Al-Shukri S, Barkin J, Herschorn S, Fianu-Jonasson A, Grise P, et al. Mirabegron as add-on treatment to solifenacin in patients with incontinent overactive bladder and an inadequate response to solifenacin monotherapy. J Urol. 2016;196(3):809-18. | 6 |
| Maman K, Aballea S, Nazir J, Desroziers K, Neine M, Siddiqui E, et al. Comparative efficacy and safety of treatments for the management of overactive bladder: A systematic literature review and mixed treatment comparison. Value Health. 2013;16(3):A180. | 5 |
| Martan A, Masata J, Krhut J, Zachoval R, Hanus T, Svabik K. Persistence in the treatment of overactive bladder syndrome (OAB) with mirabegron in a multicenter clinical study. European journal of obstetrics gynecology and reproductive biology. 2017;210:247-50. | 6 |
| Martina R, Kay R, Abrams P, van Maanen R, Ridder A. A clinical perspective on the analysis and presentation of the number of incontinence episodes following treatment for OAB. Neurourol Urodyn. 2016;35(6):728-32. | 5 |
| Mauseth SA, Skurtveit S, Spigset O. Adherence, persistence and switch rates for anticholinergic drugs used for overactive bladder in women: data from the Norwegian Prescription Database. Acta Obstetricia et Gynecologica Scandinavica. 2013;92(10):1208-15. | 2 |
| Moga DC, Carnahan RM, Lund BC, Pendergast JF, Wallace RB, Torner JC, et al. Risks and benefits of bladder antimuscarinics among elderly residents of Veterans Affairs Community Living Centers. Journal of the American Medical Directors Association. 2013;14(10):749-60. | 6 |
| Nardulli R, Losavio E, Ranieri M, Fiore P, Megna G, Bellomo RG, et al. Combined antimuscarinics for treatment of neurogenic overactive bladder. International Journal of Immunopathology & Pharmacology. 2012;25(1 Suppl):35S-41S. | 3 |
| Nazir J, Kelleher C, Aballéa S, Maman K, Hakimi Z, Mankowski C, et al. Comparative efficacy and tolerability of solifenacin 5 mg/day versus other oral antimuscarinic agents in overactive bladder: A systematic literature review and network meta‐analysis. Neurourology and urodynamics. 2017. | 8 |
| Nitti V, Herschorn S, Khullar V, Cambronero J, Angulo J, Blauwet M, et al. Efficacy of mirabegron in patients with and without prior treatment with antimuscarinic therapy for overactive bladder: Prespecified analysis of three randomised phase iii studies. International urogynecology journal and pelvic floor dysfunction.23(2 SUPPL. 1):S79-S80. | 5 |
| Obloza A, Kirby J, Yates D, Toozs‐Hobson P. Indirect treatment comparison (ITC) of medical therapies for an overactive bladder. Neurourology and urodynamics2017. p. 1824-31. | 8 |
| Olivera CK, Meriwether K, El-Nashar S, Grimes CL, Chen CC, Orejuela F, et al. Nonantimuscarinic treatment for overactive bladder: a systematic review. American Journal of Obstetrics & Gynecology. 2016;215(1):34-57. | 8 |
| Reynolds WS, McPheeters M, Blume J, Surawicz T, Worley K, Wang L, et al. Comparative Effectiveness of Anticholinergic Therapy for Overactive Bladder in Women: A Systematic Review and Meta-analysis. Obstetrics & Gynecology. 2015;125(6):1423-32. | 8 |
| Schneider T, Bergqvist A, Wang J, Wagg A, Ebel-Bitoun C. Treatment with fesoterodine versus tolterodine for reducing symptom bother in elderly patients with overactive bladder including urgency urinary incontinence. European Urology, Supplements.11(1):e687-ea. | 5 |
| Sicras-Mainar A, Rejas J, Navarro-Artieda R, Aguado-Jodar A, Ruiz-Torrejon A, Ibanez-Nolla J, et al. Antimuscarinic persistence patterns in newly treated patients with overactive bladder: a retrospective comparative analysis. Int Urogynecol J Pelvic Floor Dysfunct2014. p. 485-92. | 2 |
| Vouri SM, Kebodeaux CD, Stranges PM, Teshome BF. Adverse events and treatment discontinuations of antimuscarinics for the treatment of overactive bladder in older adults: A systematic review and meta-analysis. Arch Gerontol Geriatr. 2017;69:77-96. | 8 |
| Wagg A, Cardozo L, Nitti VW, Castro-Diaz D, Auerbach S, Blauwet MB, et al. The efficacy and tolerability of the beta3-adrenoceptor agonist mirabegron for the treatment of symptoms of overactive bladder in older patients. Age and ageing. 2014;43(5):666-75. | 6 |
| Wagg A, Dale M, Tretter R, Stow B, Compion G. Randomised, multicentre, placebo-controlled, double-blind crossover study investigating the effect of solifenacin and oxybutynin in elderly people with mild cognitive impairment: the SENIOR study. Eur Urol. 2013;64(1):74-81. | 4 |
| Wagg AS, Foley S, Peters J, Nazir J, Kool-Houweling L, Scrine L. Persistence and adherence with mirabegron vs antimuscarinics in overactive bladder: Retrospective analysis of a UK General Practice prescription database. Int J Clin Pract. 2017;71(10). | 6 |
| Wein AJ. Re: Randomised, multicentre, placebo-controlled, double-blind crossover study investigating the effect of solifenacin and oxybutynin in elderly people with mild cognitive impairment: the SENIOR study. J Urol. 2014;191(3):739-40. | 4 |
| Yang YW, Liu HH, Lin TH, Chuang HY, Hsieh T. Association between different anticholinergic drugs and subsequent dementia risk in patients with diabetes mellitus. PLoS ONE [Electronic Resource]. 2017;12(4):e0175335. | 6 |

**Table S4. Data abstraction of included studies published after 2012 review**

| **Author, Year Total Population (Included population) Country**  **Trial name (Quality rating)** | **Population** | **Interventions** | **Age, y Gender,**  **% Female Race** | **Other population characteristics**  ***(prognostic)*** |
| --- | --- | --- | --- | --- |
| Abrams et al., | Adults with | Mirabegron 25 mg (n = 77) | Age: 54.5 | Mean BMI, kg/m2: 26.5 |
| 2015 [1] | OAB | vs. |  |  |
|  | symptoms | Mirabegron 50 mg (n = 78) | Female: | Duration of OAB symptoms, months: |
|  |  | vs. | 66.5% | 58.3 |
| N = 1,306 |  | Solifenacin 5 mg (n = 156) |  |  |
| (n = 686) |  | vs. | Race: | Previous OAB drug therapy, %: 46.9% |
|  |  | Solifenacin 10 mg (n = 78) | White: |  |
| Multinational |  | vs. | 99.7% | Type of OAB, %: |
|  |  | Solifenacin 5 mg + |  | ‐Urgency incontinence: 25.7% |
| SYMPHONY |  | Mirabegron 25 mg (n = 144) |  | ‐Mixed stress/urgency incontinence: |
|  |  | vs. |  | 13.4% |
| (Fair) |  | Solifenacin 5 mg + |  | ‐Frequency/urgency incontinence: |
|  |  | Mirabegron 50 mg (n = 153) |  | 60.5% |
|  |  | Duration: 12 weeks |  | Mean incontinence episodes /24 h |
|  |  |  |  | (for patients who reported ≥ 1 |
|  |  |  |  | incontinence episode): 1.3 |
|  |  |  |  | Mean micturitions /24 h: 11.2 |
|  |  |  |  | Mean urgency episodes /24 h: 6.4 |
|  |  |  |  | Mean urgency incontinence episodes |
|  |  |  |  | /24 h: 1.3 |

| **Author, Year Total Population (Included population) Country**  **Trial name (Quality rating)** | **Efficacy/effectiveness outcomes**  ***(from PICOS; see checklist)*** | **Harms**  ***(from PICOS; see checklist)*** | **Funding/ Comments** |
| --- | --- | --- | --- |
| Abrams et al., | Mirabegron 25 mg vs. Mirabegron 50 mg vs. Solifenacin 5 mg vs. | Mirabegron 25 mg vs. Mirabegron 50 mg vs. | Astellas |
| 2015 [1] | Solifenacin 10 mg vs. Solifenacin 5 mg + Mirabegron 25 mg vs. | Solifenacin 5 mg vs. Solifenacin 10 mg vs. |  |
|  | Solifenacin 5 mg + Mirabegron 50 mg | Solifenacin 5 mg + Mirabegron 25 mg vs. Solifenacin  5 mg + Mirabegron 50 mg |  |
| N = 1,306  (n = 686) | Least squares mean (LSM) change from baseline number of incontinence episodes /24 h (SE): ‐0.74 (0.415) vs. ‐0.90 (0.353) vs. ‐0.88 (0.252) vs. ‐0.97 (0.386) vs. ‐1.22 (0.266) vs. ‐1.14 (0.306) | Withdrawal due to adverse event: 1/77 (1.3%) vs. 2/78 (2.6%) vs. 1/156 (0.6%) vs. 2/78 (2.6%) vs.  4/144 (2.8) vs. 1/153 (0.7%) |  |
| Multinational |  |  |  |
|  | LSM change from baseline number of micturitions /24 h (SE): ‐ | Serious treatment‐emergent adverse events: 0/77 |  |
| SYMPHONY | 2.48 (0.298) vs. ‐2.56 (0.296) vs. ‐2.54 (0.212) vs. ‐3.22 (0.298) vs. ‐ | (0%) vs. 2/78 (2.6%) vs. 0/156 (0%) vs. 1/78 (1.3%) |  |
|  | 2.56 (0.219) vs. ‐3.34 (0.212) | vs. 2/144 (1.4%) vs. 2/153 (1.3%) |  |
| (Fair) |  |  |  |
|  | LSM change from baseline mean number of urgency episodes |  |  |
|  | (grade ≥3) /24 h (SE): ‐3.23 (0.336) vs ‐3.44 (0.334) vs ‐2.73 |  |  |
|  | (0.239) vs ‐3.98 (0.336) vs. ‐3.86 (0.247) vs ‐4.10 (0.239) |  |  |
|  | LSM change from baseline number of urge incontinence episodes |  |  |
|  | /24 h (SE): ‐0.80 (0.424) vs .‐0.88 (0.372) vs. ‐0.86 (0.258) vs. ‐1.13 |  |  |
|  | (0.424) vs. ‐1.20 (0.277) vs. ‐1.08 (0.321) |  |  |

| **Author, Year Total Population (Included population) Country**  **Trial name (Quality rating)** | **Population** | **Interventions** | **Age, y Gender,**  **% Female Race** | **Other population characteristics**  ***(prognostic)*** |
| --- | --- | --- | --- | --- |
| Abrams et al., |  |  |  |  |
| 2015 [1] |  |  |  |  |
|  |  |  |  |  |
| continued |  |  |  |  |
| N = 1,306 |  |  |  |  |
| (n = 686) |  |  |  |  |
| Multinational |  |  |  |  |
| SYMPHONY |  |  |  |  |
| (Fair) |  |  |  |  |

| **Author, Year Total Population (Included population) Country**  **Trial name (Quality rating)** | **Efficacy/effectiveness outcomes**  ***(from PICOS; see checklist)*** | **Harms**  ***(from PICOS; see checklist)*** | **Funding/ Comments** |
| --- | --- | --- | --- |
| Abrams et al., | LSM change from baseline Overactive Bladder Questionnaire (OAB‐ | Specific adverse events: |  |
| 2015 [1] | q) Symptom Bother score (SE): ‐27.1 (2.01) vs. ‐27.5 (2.00) vs. ‐ | ‐Blurred vision: 0/77 (0%) vs. 1/78 (1.3%) vs. 0/156 |  |
| continued  N = 1,306  (n = 686) | 26.8 (1.42) vs. ‐29.9 (2.01) vs. ‐32.0 (1.48) vs. ‐33.5 (1.44)  LSM change from baseline Patient Perception of Bladder Condition (PPBC) score (SE): ‐1.4 (0.14) vs. ‐1.5 (0.14) vs. ‐1.3 (0.10) vs. ‐1.5 (0.14) vs ‐1.7 (0.10) vs. ‐1.8 (0.10)  Zero incontinence achieved EOT: 6/13 (46.2) vs. 11/18 (61.1) vs. 21/35 (60) vs. 8/15 (53.3) vs. 28/32 (87.5) vs. 21/24 (87.5) | (0%) vs. 1/78 (1.3%) vs. 2/144 (1.4%) vs. 2/153  (1.3%)  ‐Confusional state: 0/77 (0%) vs. 0/78 (0%) vs. 0/156 (0%) vs. 1/78 (1.3%) vs. 0/144 (0%) vs. 0/153 (0%)  ‐Constipation: 0/77 (0%) vs. 3/78 (3.8%) vs. 3/156 (1.9%) vs. 4/78 (5.1%) vs. 4/144 (2.8%) vs. 2/153 |  |
|  |  | (1.3%) |  |
| Multinational |  | ‐Dizziness: 1/77 (1.3%) vs. 0/78 (0%) vs. 1/156 |  |
|  |  | (0.6%) vs. 3/78 (3.8%) vs. 1/144 (0.7%) vs. 0/153 |  |
| SYMPHONY |  | (0%) |  |
|  |  | ‐Dry mouth: 2/77 (2.6%) vs. 4/78 (5.1%) vs. 18/156 |  |
| (Fair) |  | (11.5%) vs. 23/78 (29.5%) vs. 21/144 (14.6%) vs.  20/153 (13.1%) |  |
|  |  | ‐ECG QT prolonged: 0/77 (0%) vs. 0/78 (0%) vs. |  |
|  |  | 0/156 (0%) vs. 2/78 (2.6%) vs. 1/144 (0.7%) vs. |  |
|  |  | 1/153 (0.7%) |  |

| **Author, Year Total Population (Included population) Country**  **Trial name (Quality rating)** | **Population** | **Interventions** | **Age, y Gender,**  **% Female Race** | **Other population characteristics**  ***(prognostic)*** |
| --- | --- | --- | --- | --- |
| Batista et al., | Adults with | Mirabegron 50 mg (n = 936) | Age: 57.0 | Duration of OAB symptoms, months: |
| 2015 [2] | OAB | vs. |  | 61.1 |
|  | symptoms | Solifenacin 5 mg (n = 934) | Female: |  |
|  |  |  | 76.0% | Previous OAB drug therapy, %: 100% |
| N = 1,887 |  | Duration: 12 weeks |  | ‐1 antimuscarinic: 57.1% |
|  |  |  | Race: NR | ‐2 antimuscarinics: 27.9% |
| Multinational |  |  |  | ‐>2 antimuscarinics: 15.0% |
| BEYOND |  |  |  | Type of OAB, %: |
|  |  |  |  | ‐Urgency incontinence: 41.2 % |
| (Good) |  |  |  | ‐Mixed stress/urgency incontinence: |
|  |  |  |  | 15.7% |
|  |  |  |  | ‐Frequency/urgency without |
|  |  |  |  | incontinence: 43.2% |
|  |  |  |  | Mean incontinence episodes /24 h: |
|  |  |  |  | 2.1 |
|  |  |  |  | Mean micturitions /24 h: 11.5 |
|  |  |  |  | Mean urgency episodes (grade 3 or 4) |
|  |  |  |  | /24 h: 7.8 |
|  |  |  |  | Mean urgency incontinence (grade ≥3) |
|  |  |  |  | episodes /24 h: 2.0 |

| **Author, Year Total Population (Included population) Country**  **Trial name (Quality rating)** | **Efficacy/effectiveness outcomes**  ***(from PICOS; see checklist)*** | **Harms**  ***(from PICOS; see checklist)*** | **Funding/ Comments** |
| --- | --- | --- | --- |
| Batista et al., | Mirabegron 50 mg vs. Solifenacin 5 mg | Mirabegron 50 mg vs. Solifenacin 5 mg | Astellas |
| 2015 [2] |  |  |  |
|  | LSM change from baseline number of incontinence episodes /24 h | Discontinued due to adverse events: |  |
|  | (SE): ‐1.41 (0.115) vs. ‐1.66 (0.114) | 13/936 (1.4%) vs. 17/934 (1.8%) |  |
| N = 1,887 |  |  |  |
|  | Adjusted mean change from baseline number of micturitions /24 | Serious adverse events: 14/936 (1.5%) vs. |  |
| Multinational | h (SE): ‐2.95 (0.09) vs ‐3.13 (0.09) | 13/934 (1.4%) |  |
| BEYOND | LSM change from baseline number of urgency episodes (grade ≥3) | Specific adverse events: |  |
|  | /24 h (SE): ‐4.61 (0.115) vs. ‐4.84 (0.115) | ‐Blurred vision: 6/936 (0.6%) vs. 4/934 (0.4%) |  |
| (Good) |  | ‐Cardiac arrhythmia: 13/936 (1.4%) vs. 10/934 |  |
|  | LSM change from baseline number of urgency incontinence | (1.1%) |  |
|  | episodes /24 h (SE): ‐1.49 (0.047) vs. ‐1.59 (0.047) | ‐Constipation: 21/936 (2.2%) vs. 23/934 (2.5%) |  |
|  |  | ‐Dry mouth: 29/936 (3.1%) vs. 54/934 (5.8%) |  |
|  |  | ‐QT prolongation: 2/936 (0.2%) vs. 0/934 (0%) |  |
|  | Adjusted mean change from baseline OAB‐q Symptom Bother | ‐Syncope/fall/postural hypotension: 9/936 |  |
|  | score (SE): ‐28.89 (0.652) vs. ‐30.76 (0.655) | (1.0%) vs. 9/934 (1.0%) |  |
|  | Adjusted mean change from baseline PPBC score (SE): ‐1.53 |  |  |
|  | (0.042) vs. ‐1.67 (0.042)  Zero incontinence episodes achieved by EOT: 272/405 (67.3%) vs. 283/413 (68.5%) |  |  |

| **Author, Year Total Population (Included population) Country**  **Trial name (Quality rating)** | **Population** | **Interventions** | **Age, y Gender,**  **% Female Race** | **Other population characteristics**  ***(prognostic)*** |
| --- | --- | --- | --- | --- |
| Chapple et al., | Adults with | Mirabegron 50 mg (n = 812) | Age: 59.4 | Duration of OAB symptoms, months: |
| 2013 [3] | OAB for ≥ 3 | vs. |  | 85.6 |
|  | months | Tolterodine ER 4 mg (n = 812) | Female: |  |
|  |  |  | 74.0% | Previous OAB drug therapy, %: 59.1% |
| N = 2,444 |  | Duration: 12 months |  | ‐Mirabegron 50 mg: 21.0% |
| (n = 1,624) |  |  | Race: | ‐Mirabegron 100 mg: 23.4% |
|  |  |  | White: | ‐Tolterodine ER 4 mg: 14.7% |
| Multinational |  |  | 95.9% |  |
|  |  |  |  | Type of OAB, %: |
| TAURUS |  |  |  | ‐Urgency incontinence: 37.7% |
|  |  |  |  | ‐Mixed stress/urgency incontinence: |
| (Fair) |  |  |  | 27.2% |
|  |  |  |  | ‐Frequency: 35.0% |
|  |  |  |  | Mean incontinence episodes /24 h, |
|  |  |  |  | ml: 2.5 |
|  |  |  |  | Mean micturitions /24 h: 11.0 |

| **Author, Year Total Population (Included population) Country**  **Trial name (Quality rating)** | **Efficacy/effectiveness outcomes**  ***(from PICOS; see checklist)*** | **Harms**  ***(from PICOS; see checklist)*** | **Funding/ Comments** |
| --- | --- | --- | --- |
| Chapple et al., | Mirabegron 50 mg vs. Tolterodine ER 4 mg | Mirabegron 50 mg vs. Tolterodine ER 4 mg | Astellas Pharma |
| 2013 [3] |  |  |  |
|  | LSM change from baseline number of incontinence episodes /24 h | Withdrawals due to adverse events: 52/815 |  |
|  | (SE): ‐1.01 (0.087) vs. ‐1.26 (0.086) | (6.4%) vs. 49/813 (6.0%) |  |
| N = 2,444 |  |  |  |
| (n = 1,624) | LSM change from baseline number of micturitions /24 h (SE): ‐ | Serious treatment‐emergent adverse events: |  |
|  | 1.27 (0.083) vs. ‐1.39 (0.083) | 42/812 (5.2%) vs. 44/812 (5.4%) |  |
| Multinational |  |  |  |
|  | LSM change from baseline number of urgency episodes (grade ≥3) | Specific adverse events: |  |
| TAURUS | /24 h (SE): ‐1.62 (0.108) vs. ‐1.63 (0.108) | ‐Cardiac arrhythmia: 32/812 (3.9%) vs. 49/812 |  |
|  |  | (6.0%) |  |
| (Fair) | LSM change from baseline number of urgency incontinence | ‐Constipation: 23/812 (2.8%) vs. 22/812 (2.7%) |  |
|  | episodes /24 h (SE): ‐1.01 (0.082) vs. ‐1.21 (0.081) | ‐Corrected QT interval prolongation: 3/812 |  |
|  |  | (0.4%) vs. 3/812 (0.4%) |  |
|  | LSM change from baseline OAB‐q Symptom Bother score (SE): ‐ | ‐Dizziness: 22/812 (2.7%) vs. 13/820 (1.6%) vs. |  |
|  | 13.1 (0.65) vs. ‐14.3 (0.65) | 21/812 (2.6%) |  |
|  |  | ‐Dry mouth: 23/812 (2.8%) vs. 70/812 (8.6%) |  |
|  | LSM change from baseline PPBC score (SE): ‐0.8 (0.04) vs. ‐0.8 |  |  |
|  | (0.04)  Zero incontinence for 3 days per diary (%): 208/479 (43.4%) vs. 220/488 (45.1%) |  |  |

| **Author, Year Total Population (Included population) Country**  **Trial name (Quality rating)** | **Population** | **Interventions** | **Age, y Gender,**  **% Female Race** | **Other population characteristics**  ***(prognostic)*** |
| --- | --- | --- | --- | --- |
| Chapple et al., | Adults with | Mirabegron 25 mg (n = 169) | Age: 57.0 | Duration of OAB symptoms, months: |
| 2013 [4] | OAB | vs. |  | 46.8 |
|  | symptoms | Mirabegron 50 mg (n = 169) | Female: |  |
|  | for ≥3 | vs. | 87.1% | Previous OAB drug therapy, %: 46.3% |
| Astellas, 2010 [5] | months | Tolterodine 4 mg (n = 85) |  |  |
|  |  |  | Race: | Type of OAB, %: |
|  |  | Duration: 12 weeks | Caucasian | ‐Urgency incontinence: 43.9% |
|  |  |  | : 96.7% | ‐Mixed incontinence: 26.7% |
| N = 928 |  |  |  |  |
| (n = 423) |  |  |  | Mean micturitions /24 h: 12.0 |
| Multinational |  |  |  |  |
| DRAGON |  |  |  |  |
| (Fair) |  |  |  |  |

| **Author, Year Total Population (Included population) Country**  **Trial name (Quality rating)** | **Efficacy/effectiveness outcomes**  ***(from PICOS; see checklist)*** | **Harms**  ***(from PICOS; see checklist)*** | **Funding/ Comments** |
| --- | --- | --- | --- |
| Chapple et al., | Mirabegron 25 mg vs. Mirabegron 50 mg vs. Tolterodine 4 mg | Mirabegron 25 mg vs. Mirabegron 50 mg vs. | Astellas |
| 2013 [4] |  | Tolterodine 4 mg | Pharmaceuticals |
|  | Adjusted mean change from baseline number of incontinence |  |  |
|  | episodes /24 h (95% CI): ‐1.36 (‐1.45 to ‐0.23) vs. ‐1.15 (‐1.22 to ‐ | Withdrawals due to adverse events: 9/169 |  |
| Astellas, 2010 [5] | 0.02) vs. ‐0.81 (‐1.01 to 0.45) | (5.3%) vs. 4/169 (2.4%) vs. 1/85 (1.2%) |  |
|  |  |  |  |
|  | Adjusted mean change from baseline number of micturitions /24 | Serious adverse events: 1/169 (0.6%) vs. 1/169 |  |
|  | h (95% CI): ‐1.88 (‐0.99 to 0.10) vs. ‐2.08 (‐1.19 to ‐0.10) vs. ‐1.99 | (0.6%) vs. 1/85 (1.2%) |  |
| N = 928 | (‐1.18 to 0.15) |  |  |
| (n = 423) |  |  |  |
|  | Adjusted mean change from baseline number of urgency episodes |  |  |
| Multinational | (grade ≥3) /24 h (95% CI): ‐1.77 (‐1.38 to ‐0.01) vs. ‐1.67 (‐1.29 to |  |  |
|  | 0.08) vs. ‐1.46 (‐1.21 to 0.47) |  |  |
| DRAGON |  |  |  |
|  | Adjusted mean change from baseline number of urge |  |  |
| (Fair) | incontinence episodes /24 h (95% CI): ‐1.31 (‐1.38 to ‐0.35) vs. ‐ |  |  |
|  | 1.13 (‐1.18 to ‐0.19) vs. ‐0.76 (‐0.92 to 0.30) |  |  |
|  | Adjusted change from baseline International Consultation on |  |  |
|  | Incontinence Modular Questionnaire (ICIQ) overactive bladder |  |  |
|  | score (95% CI): ‐2.40 (‐1.13 to ‐0.02) vs. ‐2.51(‐1.24 to ‐0.13) vs. ‐ |  |  |
|  | 2.21 (‐1.05 to 0.31) |  |  |
|  |  |  |  |
|  | Zero incontinence for 3 days per diary (%): NR vs. 45/108 (41.7) vs. 19 |  |  |
|  | 19/53 (35.85) |  |  |

| **Author, Year Total Population (Included population) Country**  **Trial name (Quality rating)** | **Population** | **Interventions** | **Age, y Gender,**  **% Female Race** | **Other population characteristics**  ***(prognostic)*** |
| --- | --- | --- | --- | --- |
| Drake et al., | Adults with | Solifenacin 5 mg + | Age: 57.4 | Mean BMI, kg/m2: 29.0 |
| 2016 [6] | OAB for ≥3 | Mirabegron 25 mg (increasing |  |  |
|  | months with | to 50 mg after 4 weeks) (n = | Female: | Duration of OAB symptoms, months: |
|  | ≥1 | 727) | 83.3% | 71.2 |
| N = 2,174 | incontinence | vs. |  |  |
|  | episode | Solifenacin 5 mg (n = 728) | Race: | Previous OAB medication, %: 68.2% |
| Multinational | /24 h after 4 | vs. | White: | ‐1 previous OAB medication: 37.6% |
|  | weeks of | Solifenacin 10 mg (n = 719) | 94.2% | ‐2 previous OAB medications: 17.0% |
| BESIDE | treatment |  | Black: | ‐>2 previous OAB medications: 13.6% |
|  | with | Duration: 12 weeks | 3.3% | ‐Solifenacin: 40.1% |
| (Good) | solifenacin 5 |  | Asian: | ‐Mirabegron: 5.8% |
|  | mg |  | 2.0% |  |
|  |  |  | Other: | Mean incontinence episodes /24 h: |
|  |  |  | 0.4% | 3.23 |
|  |  |  |  | Mean micturitions /24 h: 9.00 |
|  |  |  |  | Mean urgency episodes /24 h: 5.77 |
|  |  |  |  | Mean urgency incontinence episodes |
|  |  |  |  | /24 h: 2.94 |

| **Author, Year Total Population (Included population) Country**  **Trial name (Quality rating)** | **Efficacy/effectiveness outcomes**  ***(from PICOS; see checklist)*** | **Harms**  ***(from PICOS; see checklist)*** | **Funding/ Comments** |
| --- | --- | --- | --- |
| Drake et al., | Mirabegron 25‐50 mg + Solifenacin 5 mg vs. Solifenacin 5 mg vs. | Mirabegron 25‐50 mg + Solifenacin 5 mg vs. | Astellas |
| 2016 [6] | Solifenacin 10 mg | Solifenacin 5 mg vs. Solifenacin 10 mg |  |
|  |  |  |  |
|  | Adjusted mean change from baseline number of incontinence | Withdrawal to adverse events: 11/725 (1.5%) |  |
| N = 2,174 | episodes /24 h (SE): ‐1.8 (0.08) vs. ‐1.53 (0.08) vs. ‐1.67 (0.08) | vs. 11/728 (1.5%) vs. 11/719 (1.5%) |  |
| Multinational | LSM change from baseline number of micturitions /24 h (SE): ‐ | Serious treatment‐emergent adverse events: |  |
|  | 2.10 (0.13) vs. ‐2.58 (0.97) vs. ‐2.16 (0.13) | 13/725 (1.8%) vs. 10/728 (1.4%) vs. 15/719 |  |
| BESIDE |  | (2.1%) |  |
|  | Adjusted mean change from baseline number of urgency episodes |  |  |
| (Good) | (grade ≥3) /24 h (SE): ‐2.95 (0.10) vs. ‐2.41 (0.10) vs. ‐2.54 (0.11) | Specific adverse events: |  |
|  |  | ‐Constipation: 33/725 (4.6%) vs. 22/728 (3.0) |  |
|  | Adjusted mean change from baseline number of urgency | vs. 34/719 (4.7%) |  |
|  | incontinence episodes /24 h (SE): ‐1.82 (0.07) vs. ‐1.54 (0.07) vs. ‐ | ‐Dizziness: 6/725 (0.8%) vs. 11/728 (1.5%) vs. |  |
|  | 1.63 (0.07) | 8/719 (1.1%) |  |
|  |  | ‐Dry mouth: 43/725 (5.9%) vs. 41/728 (5.6%) |  |
|  | LSM change from baseline OAB‐q Symptom Bother score (SE): ‐ | vs. 68/719 (9.5%) |  |
|  | 26.89 (0.69) vs. ‐21.93 (0.70) vs. ‐23.59 (0.70) | ‐Increased heart rate, tachycardia, atrial |  |
|  |  | fibrillation, and palpitations: 7/725 (10%) vs. |  |
|  | Adjusted mean change from baseline PPBC score (SE): ‐1.5 (0.0) | 5/728 (0.7%) vs. 4/719 (0.6%) |  |
|  | vs. ‐1.2 (0.0) vs. ‐1.3 (0.0)  Zero incontinence achieved at EOT (%): 325/706 (46.0) vs. 267/704 (37.9) vs. 280/697 (40.2) | ‐QT prolongation: 1/725 (0.1%) vs. 1/728 |  |
|  |  | (0.1%) vs. 2/719 (0.3%) |  |

| **Author, Year Total Population (Included population) Country**  **Trial name (Quality rating)** | **Population** | **Interventions** | **Age, y Gender,**  **% Female Race** | **Other population characteristics**  ***(prognostic)*** |
| --- | --- | --- | --- | --- |
| Ercan et al., 2015 [7]  N = 119  Turkey (Fair) | Adults with OAB  symptoms | Solifenacin 5 mg (n = 60) vs.  Fesoterodine 4 mg (n = 59)  Duration: 12 weeks | Age: 58.5  Female: 100%  Race: NR | Mean BMI, kg/m2: 27.1  Duration of OAB symptoms, months: 17 |

| **Author, Year Total Population (Included population) Country**  **Trial name (Quality rating)** | **Efficacy/effectiveness outcomes**  ***(from PICOS; see checklist)*** | **Harms**  ***(from PICOS; see checklist)*** | **Funding/ Comments** |
| --- | --- | --- | --- |
| Ercan et al., 2015 [7]  N = 119  Turkey (Fair) | Solifenacin 5 mg vs. Fesoterodine4 mg  Change from baseline Overactive Bladder Symptom Scores (OABSS): ‐8.2 vs. ‐9.4 | Solifenacin 5 mg vs. Fesoterodine 4 mg  Discontinuation due to adverse events: 0/60 (0%) vs. 6/59 (10.2%)  Serious adverse events: NR Specific adverse events:  ‐Constipation: 1/60 (1.7%) vs. 3/59 (5.1%)  ‐Dry mouth: 3/60 (5.0%) vs. 8/59 (13.6%) | NR |

| **Author, Year Total Population (Included population) Country**  **Trial name (Quality rating)** | **Population** | **Interventions** | **Age, y Gender,**  **% Female Race** | **Other population characteristics**  ***(prognostic)*** |
| --- | --- | --- | --- | --- |
| Gratzke et al., | Adults with | Mirabegron 50 mg (n = 306) | Age: 58.5 | Mean BMI, kg/m2: 28.8 |
| 2018 [8] | urinary | vs. |  |  |
|  | frequency | Solifenacin 5 mg + | Female: | Duration of "wet" OAB symptoms, |
|  | and urgency | Mirabegron 50 mg (n = 1218) | 79.9% | months: 73.5 |
| N=1,829 | with | vs. |  |  |
|  | incontinence | Solifenacin 5 mg (n = 305) | Race: | Previous OAB drug therapy, %: 46.4% |
| Multinational | for ≥3 |  | White: | ‐Mirabegron: 4.6% |
|  | months | Duration: 12 months | 87.1% | ‐Solifenacin: 25.3% |
| SYNERGY II |  |  | Black: |  |
|  |  |  | 2.0% | Type of OAB, %: |
| (Good) |  |  | Asian: | ‐Urgency incontinence only: 72.2% |
|  |  |  | 10.1% | ‐Mixed stress/urgency incontinence: |
|  |  |  | Other: 0.7 | 27.8% |
|  |  |  | % |  |
|  |  |  |  | Mean incontinence episodes /24 h: |
|  |  |  |  | 3.1 |
|  |  |  |  | Mean micturitions /24 h: 10.6 |
|  |  |  |  | Mean urgency urinary incontinence |
|  |  |  |  | episodes /24 h: 2.9 |

| **Author, Year Total Population (Included population) Country**  **Trial name (Quality rating)** | **Efficacy/effectiveness outcomes**  ***(from PICOS; see checklist)*** | **Harms**  ***(from PICOS; see checklist)*** | **Funding/ Comments** |
| --- | --- | --- | --- |
| Gratzke et al., | Mirabegron 50 mg vs. Solifenacin 5 mg + Mirabegron 50 mg vs. | Mirabegron 50 mg vs. Solifenacin 5 mg + | Astellas Pharma |
| 2018 [8] | Solifenacin 5mg | Mirabegron 50 mg vs. Solifenacin 5mg | Europe |
|  |  |  |  |
|  | Adjusted mean change from baseline number of incontinence | Withdrawals due to treatment‐emergent |  |
| N=1,829 | episodes /24 h (SE): ‐1.6 (0.1) vs. ‐2.0 (0.1) vs. ‐1.9 (0.1) | adverse events: 7/305 (2.3%) vs. 25/1206 |  |
|  |  | (2.1%) vs. 5/303 (1.7%) |  |
| Multinational | Adjusted mean change from baseline number of micturitions /24 |  |  |
|  | h (SE): ‐2.1 (0.1) vs. ‐2.6 (0.1) vs. ‐2.2 (0.1) | Serious treatment‐emergent adverse event: |  |
| SYNERGY II |  | 8/305 (2.6%) vs. 51/1206 (4.2%) vs. 8/303 |  |
|  | LSM change from baseline number of urgency episodes (grade ≥ | (2.6%) |  |
| (Good) | 3) /24 h (SE): ‐3.11 (0.17) vs. ‐3.84 (0.08) vs .‐3.345 (0.17) |  |  |
|  |  | Specific treatment‐emergent adverse events: |  |
|  | LSM change from baseline number of urgency incontinence | ‐Constipation: 3/305 (1.0%) vs.40/1206 (3.3%) |  |
|  | episodes /24 h (SE): ‐1.82 (0.07) vs. ‐1.54 (0.07) vs. 01.63 (0.07) | vs. 7/303 (2.3%) |  |
|  |  | ‐Dizziness: 4/305 (1.3%) vs. 13/1206 (1.1%) vs. |  |
|  | LSM change from baseline number of pads used /24 h (SE): ‐1.23 | 0/303 (0%) |  |
|  | (0.12) vs. ‐1.66 (0.06) vs. ‐1.38 (0.12) | ‐Dry mouth: 12/305 (3.9%) vs. 74/1206 (6.1%) |  |
|  |  | vs. 18/303 (5.9%) |  |
|  | Adjusted mean change from baseline OAB‐q symptom bother | ‐QT interval prolongation: 3/305 (1.0%) vs. |  |
|  | score (SE): ‐22.0 (1.1) vs. ‐29.5 (0.6) vs. ‐24.9 (1.1) | 3/1206 (0.2%) vs. 0/303 (0%) |  |
|  |  | ‐Tachyarrhythmia: 8/305 (2.6%) vs. 36/1206 |  |
|  | LSM change from baseline PPBC score (SE): ‐1.22 (0.07) vs. ‐1.54 | (3.0%) vs. 3/303 (1.0%) |  |
|  | (0.04) vs. ‐1.34 (0.07)  Zero incontinence episodes/24h at EOT: 144/301 (47.8%) vs. 696/1184 (58.8%) vs. 158/297 (53.2%) | ‐Tachycardia: 5/305 (1.6%) vs. 23/1206 (1.9%) |  |
|  |  | vs. 1/303 (0.3%) |  |

| **Author, Year Total Population (Included population) Country**  **Trial name (Quality rating)** | **Population** | **Interventions** | **Age, y Gender,**  **% Female Race** | **Other population characteristics**  ***(prognostic)*** |
| --- | --- | --- | --- | --- |
| Herschorn et al., | Adults with | Mirabegron 25 mg (n = 441) | Age: 57.3 | Mean BMI, kg/m2: 28.4 |
| 2017 [9] | "wet" OAB | vs. |  |  |
|  | symptoms | Mirabegron 50 mg (n = 437) | Female: | Duration of wet OAB symptoms, |
|  |  | vs. | 77.1% | months: 66.8 |
| Robinson et al., |  | Solifenacin 5 mg (n = 434) |  |  |
| 2017 [10] |  | vs. | Race: | Previous OAB drug therapy, %: 46.2% |
|  |  | Solifenacin 5mg + mirabegron | White: | ‐Solifenacin: 23.0% |
|  |  | 25mg (n = 885) | 79.5% | ‐Mirabegron: 3.9% |
| White et al., |  | vs. | Black: |  |
| 2018 [11] |  | Solifenacin 5 mg + | 3.4% | Type of OAB, %: |
|  |  | Mirabegron 50 mg (n = 883) | Asian: | ‐Urgency incontinence: 65.3% |
|  |  |  | 15.1% | ‐Mixed stress/urgency incontinence: |
| N = 3,527 |  | Duration: 12 weeks | Other: | 34.7% |
| (n = 3,080) |  |  | 1.4% |  |
|  |  |  | Unknown: | Mean incontinence episodes /24 h: |
| Multinational |  |  | 0.6% | 3.28 |
| SYNERGY |  |  |  | Mean micturitions /24 h: 10.81 |
| (Fair) |  |  |  | Mean urgency (grade ≥3) episodes /24 |
|  |  |  |  | h: 6.29 |
|  |  |  |  | Mean urgency incontinence episodes |
|  |  |  |  | /24 h: 2.92 |

| **Author, Year Total Population (Included population) Country**  **Trial name (Quality rating)** | **Efficacy/effectiveness outcomes**  ***(from PICOS; see checklist)*** | **Harms**  ***(from PICOS; see checklist)*** | **Funding/ Comments** |
| --- | --- | --- | --- |
| Herschorn et al., | Mirabegron 25 mg vs. Mirabegron 50 mg vs. Solifenacin 5 mg vs. | Mirabegron 25 mg vs. Mirabegron 50 mg vs. | Astellas Pharma |
| 2017 [9] | Solifenacin 5mg + Mirabegron 25mg vs. Solifenacin 5 mg + Mirabegron 50 mg | Solifenacin 5 mg vs. Solifenacin 5mg + Mirabegron  25mg vs. Solifenacin 5 mg + Mirabegron 50 mg | Europe B.V. and Astellas Pharma |
| Robinson et al., | Adjusted mean change from baseline number of incontinence | Discontinuation due to adverse events: 7/423 (1.7%) vs. 10/422 (2.4%) vs. 7/423 (1.7%) vs 20/853 (2.3%) vs. | Global Development, Inc. |
| 2017 [10] | episodes /24 h (SE): ‐1.70 (0.10) vs. ‐1.76 (0.10) vs. ‐1.79 (0.10) vs. ‐ | 22/848 (2.6%) |  |
|  | 2.04 (0.07) vs. ‐1.98 (0.07) |  |  |
|  |  | Serious treatment‐emergent adverse events: 6/423 |  |
| White et al., 2018 [11] | Adjusted mean change from baseline number of micturitions / 24 h (SE): ‐2.00 (0.12) vs. ‐2.03 (0.12) vs. ‐2.20 (0.12) vs. ‐2.49 (0.08) | (1.4%) vs. 5/422 (1.2%) vs. 3/423 (0.7%) vs. 12/853  (1.4%) vs. 19/848 (2.2%) |  |
|  | vs. ‐2.59 (0.08) | Specific adverse events: |  |
|  |  | ‐Atrial fibrillation: 1/423 (0.2%) vs. 0/422 (0%) vs. |  |
| N = 3,527 | LSM change from baseline number of urgency episodes (grade ≥3) | 0/423 (0%) vs. 1/853 (0.1%) vs. 4/848 (0.5%) |  |
| (n = 3,080) | /24 h (SE): ‐2.74 (0.15) vs. ‐2.63 (0.15) vs. ‐3.05 (0.15) vs. ‐3.38 | ‐Atrial flutter: 0/423 (0%) vs. 0/422 (0%) vs. 0/423 (0%) |  |
| Multinational | (0.11) vs. ‐3.51 (0.11)  LSM change from baseline number of urgency incontinence | vs. 0/853 (0%) vs. 1/848 (0.1%)  ‐Blurred vision: 1/423 (0.2%) vs. 0/422 (0%) vs. 2/423 (0.5%) vs. 5/853 (0.6%) vs. 6/848 (0.7%)  ‐Constipation: 6/423 (1.4%) vs. 11/422 (2.6%) vs. |  |
| SYNERGY | episodes /24 h (SE): ‐1.58 (0.09) vs. ‐1.62 (0.09) vs. ‐1.71 (0.09) vs. ‐ | 6/423 (1.4%) vs. 38/853 (4.5%) vs. 31/848 (3.7%) |  |
|  | 1.95 (0.06) vs .‐1.94 (0.06) | ‐Dry mouth: 17/423 (4.0%) vs. 14/422 (3.3%) vs. |  |
| (Fair) |  | 25/423 (5.9%) vs. 74/853 (8.7%) vs. 61/848 (7.2%) |  |
|  | LSM change from baseline OAB‐q Symptom Bother score (SE): ‐ 23.93 (0.99) vs. ‐26.14 (0.98) vs. ‐26.44 (0.98) vs. ‐31.06 (0.69) vs. ‐  32.24 (0.70) | ‐Major Adverse CV Events: 0/423 (0%) vs. 0/422 (0%)  vs. 0/423 (0%) vs. 1/853 (0.1%) vs. 2/848 (0.24%)  ‐QTc prolongation: 0/423 (0%) vs. 0/422 (0%) vs. 0/423 (0%) vs. 1/853 (0.1%) vs. 2/848 (0.2%) |  |
|  |  | ‐Tachycardia: 5/423 (1.2%) vs. 6/422 (1.4%) vs. 2/423 |  |
|  | LSM change from baseline PPBC score (SE): ‐1.18 (0.06) vs. ‐1.31 | (0.5%) vs. 17/853 (2.0%) vs. 11/848 (1.3%) |  |
|  | (0.06) vs. ‐1.27 (0.06) vs. ‐1.53 (0.04) vs. ‐1.66 (0.04)  Zero incontinence for 3 days per diary: 166/409 (40.6) vs. 188/406 (46.3) vs. 177/413 (42.9) vs. 417/823 (50.7) vs. 426/816 (52.2) |  |  |

| **Author, Year Total Population (Included population) Country**  **Trial name (Quality rating)** | **Population** | **Interventions** | **Age, y Gender,**  **% Female Race** | **Other population characteristics**  ***(prognostic)*** |
| --- | --- | --- | --- | --- |
| Jafarabadi et al., | Female | Oxybutynin IR 5 mg (n = 151) | Age: 54.7 | Mean BMI, kg/m²: 27.8 |
| 2015 [12] | adults (≥45 | vs. |  |  |
|  | years) with | Tolterodine IR 4 mg (n = 150) | Female: | Mean moderate and severe |
|  | overactive |  | 100% | incontinence episodes: 8.9 |
| N = 301 | bladder | Duration: 12 weeks |  |  |
|  | syndrome |  | Race: | Mean urgency episodes: 5.9 |
| Iran |  |  | White: |  |
|  |  |  | 100% |  |
| (Fair) |  |  |  |  |

| **Author, Year Total Population (Included population) Country**  **Trial name (Quality rating)** | **Efficacy/effectiveness outcomes**  ***(from PICOS; see checklist)*** | **Harms**  ***(from PICOS; see checklist)*** | **Funding/ Comments** |
| --- | --- | --- | --- |
| Jafarabadi et al., | Oxybutynin IR 5 mg vs. Tolterodine IR 4 mg | Oxybutynin IR 5 mg vs. Tolterodine IR 4 mg | NR |
| 2015 [12] |  |  |  |
|  | Mean change in moderate and severe incontinence episodes: ‐ | Withdrawals due to adverse events: 11/151 |  |
|  | 3.58 vs. ‐3.50 (p = 0.75) | (7.3%) vs. 8/150 (5.3%) |  |
| N = 301 |  |  |  |
|  | Mean change in urgency episodes: ‐1.86 vs. ‐1.62 (p = 0.64) | Serious adverse events: NR |  |
| Iran |  |  |  |
|  |  | Specific adverse events: |  |
| (Fair) |  | ‐Dry mouth: 5/151 (3%) vs. 4/150 (2.8%); p = |  |
|  |  | 0.85 |  |

| **Author, Year Total Population (Included population) Country**  **Trial name (Quality rating)** | **Population** | **Interventions** | **Age, y Gender,**  **% Female Race** | **Other population characteristics**  ***(prognostic)*** |
| --- | --- | --- | --- | --- |
| Khullar et al., | Adults with | Mirabegron 50 mg (n = 497) | Age: 59.1 | Mean BMI, kg/m²: 27.7 |
| 2013 [13] | OAB for ≥ 3 | vs. | years |  |
|  | months | Tolterodine ER 4 mg (n = 495) |  | Duration of OAB symptoms, months: |
| Khullar et al., |  |  | Female: | 77.5 |
| 2016 [14] |  | Duration: 12 weeks | 72.3% |  |
|  |  |  |  | Previous OAB drug therapy, %: 49.7% |
|  |  |  | Race: |  |
| N = 1,987 |  |  | White: | Type of OAB, %: |
| (n = 992) |  |  | 99.1% | ‐Urgency incontinence: 39.7% |
|  |  |  | Black or | ‐Frequency: 37.9% |
| Europe, Australia |  |  | African | ‐Mixed: 22.5% |
|  |  |  | American: |  |
| SCORPIO |  |  | 0.34% |  |
|  |  |  | Asian: |  |
| (Good) |  |  | 0.40% |  |
|  |  |  | Other: |  |
|  |  |  | 0.20% |  |

| **Author, Year Total Population (Included population) Country**  **Trial name (Quality rating)** | **Efficacy/effectiveness outcomes**  ***(from PICOS; see checklist)*** | **Harms**  ***(from PICOS; see checklist)*** | **Funding/ Comments** |
| --- | --- | --- | --- |
| Khullar et al., | Mirabegron 50 mg vs. Tolterodine ER 4 mg | Mirabegron 50 mg vs. Tolterodine ER 4 mg | Astellas Pharma |
| 2013 [13] |  |  | Global |
|  | Adjusted mean change from baseline number of incontinence | Withdrawals due to adverse events: 25/497 |  |
| Khullar et al., | episodes /24 h (SE): ‐1.57 (0.113) vs. ‐1.27 (0.112) | (5.0%) vs. 24/495 (4.8%) |  |
| 2016 [14] |  |  |  |
|  | Adjusted mean change from baseline number of micturitions /24 | Serious adverse events: 14/493 (2.8%) vs. |  |
|  | h (SE); ‐1.93 (0.111) vs. ‐1.59 (0.111) | 11/495 (2.2%) |  |
| N = 1,987 |  |  |  |
| (n = 992) | LSM change from baseline number of urgency episodes (grade ≥3) | Specific adverse events (TEAE): |  |
|  | /24 h (SE): ‐2.25 (0.152) vs. ‐2.07 (0.152) | ‐Atrial fibrillation of medical importance: |  |
| Europe, Australia |  | 2/493 (0.4%) vs. 5/495 (1.0%) |  |
|  | Adjusted mean change from baseline OAB‐q Symptom Bother | ‐Constipation: 8/493 (1.6%) vs. 8/496 (1.6%) |  |
| SCORPIO | score (SE): ‐19.6 (0.85) vs. ‐18.4 (0.85) | vs. 10/495 (2.0%) |  |
|  |  | ‐Dry mouth: 14/493 (2.8%) vs. 50/495 (10.1%) |  |
| (Good) | Adjusted mean change from baseline PPBC score (SE): ‐1.0 (0.06) | ‐QTc prolongation: 0/93 (0%) vs. 2/495 (0.4%) |  |
|  | vs. ‐1.0 (0.06)  Zero incontinence for 3 days per diary (%): 132/293 (45.1) vs. 142/300 (47.3) |  |  |

| **Author, Year Total Population (Included population) Country**  **Trial name (Quality rating)** | **Population** | **Interventions** | **Age, y Gender,**  **% Female Race** | **Other population characteristics**  ***(prognostic)*** |
| --- | --- | --- | --- | --- |
| Kuo et al., 2015 | Adults with | Mirabegron 50 mg (n = 372) | Age: 54.1 | Mean weight, kg: 61.8 |
| [15] | OAB for ≥ 3 | vs. |  |  |
|  | months | Tolterodine ER 4 mg (n = 377) | Gender: | Duration of OAB symptoms, months: |
| N =1,126 |  |  | Female: | 60.0 |
| (n = 749) |  | Duration: 12 weeks | 65.7% |  |
|  |  |  |  | Previous OAB drug therapy, %: 51.7% |
| Multinational |  |  | Race: NR |  |
|  |  |  |  | Type of OAB, %: |
| (Fair) |  |  |  | ‐No incontinence: 42.6% |
|  |  |  |  | ‐Urgency incontinence: 38.7% |
|  |  |  |  | ‐Mixed stress/urgency incontinence: |
|  |  |  |  | 18.6% |
|  |  |  |  | Mean incontinence episodes/24 |
|  |  |  |  | hours: 2.3 |
|  |  |  |  | Mean micturitions /24 h: 12.1 |
|  |  |  |  | Mean urgency episodes /24 h: 5.3 |
|  |  |  |  | Mean urgency incontinence episodes |
|  |  |  |  | /24 h: 1.8 |

| **Author, Year Total Population (Included population) Country**  **Trial name (Quality rating)** | **Efficacy/effectiveness outcomes**  ***(from PICOS; see checklist)*** | **Harms**  ***(from PICOS; see checklist)*** | **Funding/ Comments** |
| --- | --- | --- | --- |
| Kuo et al., 2015 | Mirabegron 50 mg vs. Tolterodine ER 4 mg | Mirabegron 50 mg vs. Tolterodine ER 4 mg | Astellas Inc. |
| [15] |  |  |  |
|  | Change from baseline number of incontinence episodes /24 h | Discontinuation due to adverse events: 9/366 |  |
| N =1,126 | (SD): ‐1.23 (2.764) vs. ‐1.15 (1.914) | (2.5%) vs. 11/371 (3.0%) |  |
| (n = 749) |  |  |  |
|  | Change from baseline number of micturitions /24 h (SD): ‐2.12 | Serious adverse events: 5/366 (1.4%) vs. 6/371 |  |
| Multinational | (3.022) vs. ‐1.54 (2.943) | (1.6%) |  |
| (Fair) | Change from baseline number of urgency episodes (grade ≥3) /24 | Specific adverse events: |  |
|  | h (SD): ‐2.32 (3.725) vs. ‐2.43 (3.700) | ‐Constipation: 8/366 (2.2%) vs. 9/371 (2.4%) |  |
|  |  | ‐Dizziness: 5/366 (1.4%) vs. 8/371 (2.2%) |  |
|  | Change from baseline King's Health Questionnaire ‐ Bladder | ‐Dry mouth: 18/366 (4.9%) vs. 30/371 (8.1%) |  |
|  | Problem score (SD): ‐10.13 (15.01) vs. ‐10.17 (15.13) | ‐EKG QT prolonged: 1/366 (0.3%) vs. 4/371 |  |
|  |  | (1.1%) |  |

| **Author, Year Total Population (Included population) Country**  **Trial name (Quality rating)** | **Population** | **Interventions** | **Age, y Gender,**  **% Female Race** | **Other population characteristics**  ***(prognostic)*** |
| --- | --- | --- | --- | --- |
| Manjunatha et | Adults (age | Darifenacin 7.5 mg (n = 30) | Age: 64.1 | Mean BMI, kg/m2: 24.4 |
| al., 2015 [16] | between 50 ‐ | vs. |  |  |
|  | 80 years | Trospium ER 60 mg (n = 30) | Female: |  |
|  | old) with |  | 23.3% |  |
| N = 60 | AOB | Duration: 4 weeks |  |  |
|  | symptoms |  | Race: |  |
| India |  |  | Asian: |  |
|  |  |  | 100% |  |
| (Fair) |  |  |  |  |

| **Author, Year Total Population (Included population) Country**  **Trial name (Quality rating)** | **Efficacy/effectiveness outcomes**  ***(from PICOS; see checklist)*** | **Harms**  ***(from PICOS; see checklist)*** | **Funding/ Comments** |
| --- | --- | --- | --- |
| Manjunatha et | Darifenacin 7.5 mg vs. Trospium ER 60 mg | Darifenacin 7.5 mg vs. Trospium ER 60 mg | NR |
| al., 2015 [16] |  |  |  |
|  | Change from baseline OABSS composite score (SD): ‐5.80 (3.99) | Withdrawals due to adverse events: 0/30 (0%) |  |
|  | vs. ‐5.27 (2.98) | vs. 0/30 (0%) |  |
| N = 60 |  |  |  |
|  | Change from baseline OABSS frequency score (SD): ‐0.80 (0.76) vs. | Serious adverse events: NR |  |
| India | ‐0.47 (0.63) |  |  |
|  |  | Specific adverse events: |  |
| (Fair) | Change from baseline OABSS urgency score (SD): ‐1.87 (2.22) vs. ‐ | ‐Mean change in McMillan & Williams |  |
|  | 2.40 (2.19) | Constipation Assessment Scale (CAS): 0.93 vs. |  |
|  |  | 0.60 (P = 0.944) |  |
|  | Change from baseline OABSS urge urinary incontinence score | ‐Mean change in Bristol stool form scale: ‐0.13 |  |
|  | (SD): ‐2.27 (1.72) vs. ‐1.47 (1.96) | vs. 0.13 (P = 0.017) |  |
|  |  | ‐Mean change in Knowles‐Eccersley‐Scott‐ |  |
|  |  | Symptom (KESS) questionnaire score: 1.54 vs. |  |
|  |  | 0.67 (P = 0.244) |  |

| **Author, Year Total Population (Included population) Country**  **Trial name (Quality rating)** | **Population** | **Interventions** | **Age, y Gender,**  **% Female Race** | **Other population characteristics**  ***(prognostic)*** |
| --- | --- | --- | --- | --- |
| Staskin et al., | Treatment‐ | Mirabegron 50 mg (n = 316) | Age: 53.6 | Mean BMI, kg/m2: 29.7 |
| 2018 [17] | naïve adults | vs. |  |  |
|  | with OAB | Tolterodine ER 4 mg (n = 310) | Female: | Duration of OAB symptoms, months: |
| Herschorn et al., | for ≥3 |  | 73.2% | 77.3 |
| 2018 [18] | months | Duration: 18 weeks |  |  |
|  |  | (crossover: 8 weeks first | Race: | Previous OAB drug therapy, %: 0% |
|  |  | treatment, 2 weeks washout, | White: |  |
|  |  | 8 weeks second treatment) | 80.2% | Type of OAB, %: |
| N = 358 |  |  | Black: | ‐Urgency incontinence: 40.8% |
|  |  |  | 16.8% | ‐Mixed stress/urgency incontinence: |
| U.S. and Canada |  |  | Asian: | 33.8% |
|  |  |  | 2.2% | ‐Frequency/urgency without |
| PREFER |  |  | American | incontinence: 25.4% |
|  |  |  | Indian/Ala |  |
| (Fair) |  |  | ska | Mean incontinence episodes /24 h: |
|  |  |  | Native: | 3.27 |
|  |  |  | 0.3% |  |
|  |  |  | Other: | Mean micturitions /24 h: 11.58 |
|  |  |  | 0.6% |  |
|  |  |  |  | Mean urgency (grade ≥3) episodes /24 |
|  |  |  |  | h: 5.59 |
|  |  |  |  | Mean urgency incontinence episodes |
|  |  |  |  | /24 h: 2.95 |

| **Author, Year Total Population (Included population) Country**  **Trial name (Quality rating)** | **Efficacy/effectiveness outcomes**  ***(from PICOS; see checklist)*** | **Harms**  ***(from PICOS; see checklist)*** | **Funding/ Comments** |
| --- | --- | --- | --- |
| Staskin et al., | Mirabegron 50 mg vs. Tolterodine ER 4 mg | Mirabegron 50 mg vs. Tolterodine ER 4 mg | Astellas Pharma |
| 2018 [17] |  |  | Inc. |
|  | Adjusted mean change from baseline incontinence episodes /24 h | Withdrawals due to treatment‐emergent |  |
|  | (SE): ‐1.51 (0.19) vs. ‐1.46 (0.19) | adverse events: 15/319 (4.7%) vs. 20/325 |  |
| Herschorn et al., |  | (6.2%); RR 0.76 (95% CI 0.40 to 1.47) |  |
| 2018 [18] | Adjusted mean change from baseline number of micturitions /24 |  |  |
|  | h (SE): ‐2.06 (0.19) vs. ‐1.95 (0.20) | Serious treatment‐emergent adverse events: |  |
|  |  | 3/319 (0.9%) vs. 8/325 (2.5%); RR 0.38 (95% CI |  |
| N = 358 | LSM change from baseline number of urgency episodes (grade ≥3) | 0.10 to 1.43) |  |
|  | / 24 h (SE): ‐2.26 (0.19) vs. ‐2.13 (0.19) |  |  |
| U.S. and Canada |  | Specific adverse events: |  |
|  | Adjusted mean change from baseline urgency incontinence | ‐Atrial fibrillation: 2/319 (0.6%) vs. 1/325 |  |
| PREFER | episodes /24 h (SE): ‐1.42 (0.20) vs. ‐1.37 (0.20) | (0.3%) |  |
|  |  | ‐Blurred vision: 12/319 (3.8%) vs. 11/325 |  |
| (Fair) | Adjusted mean change from baseline OAB‐q Symptom Bother | (3.4%); RR 1.11 (95% CI 0.50 to 2.48) |  |
|  | score (95% CI): ‐22.32 (‐25.13 to ‐19.52) vs. ‐20.88 (‐23.69 to ‐ | ‐Common anticholinergic adverse events: |  |
|  | 18.06) | 65/319 (20.4%) vs. 89/325 (274%); RR 0.74 |  |
|  |  | (95% CI 0.56 to 0.98) |  |
|  | Adjusted mean change from baseline PPBC score (95% CI): ‐1.04 (‐ | ‐Constipation: 18/319 (5.6%) vs. 20/325 |  |
|  | 1.21 to ‐0.88) vs. ‐0.99 (‐1.16 to ‐0.83)  Zero incontinence for 3 days per diary (%): 177/255 (45.9) vs. 114/252 (45.5) | (6.2%); RR 0.92 (95% CI 0.49 to 1.70) |  |
|  |  | ‐Dry mouth: 29/319 (9.1%) vs. 53/325 (16.3%); |  |
|  |  | RR 0.56 (95% CI 0.36 to 0.85) |  |
|  |  | ‐Palpitations: 0/319 (0%) vs. 2/325 (0.6%) |  |
|  |  | ‐Tachycardia: 1/319 (0.3%) vs. 2/325 (0.6%) |  |

| **Author, Year Total Population (Included population) Country**  **Trial name (Quality rating)** | **Population** | **Interventions** | **Age, y Gender,**  **% Female Race** | **Other population characteristics**  ***(prognostic)*** |
| --- | --- | --- | --- | --- |
| Vecchioli et al., 2016 [19]  N = 80  Italy (Fair) | Adult females diagnosed with OAB syndrome | Mirabegron 50 mg (n = 40) vs.  Solifenacin 5 mg (n = 40)  Duration: 12 weeks | Age: 57  Female: 100%  Race: NR | NR |

| **Author, Year Total Population (Included population) Country**  **Trial name (Quality rating)** | **Efficacy/effectiveness outcomes**  ***(from PICOS; see checklist)*** | **Harms**  ***(from PICOS; see checklist)*** | **Funding/ Comments** |
| --- | --- | --- | --- |
| Vecchioli et al., | Mirabegron 50 mg vs. Solifenacin 5 mg | Mirabegron 50 mg vs. Solifenacin 5 mg | Operating Unit of |
| 2016 [19] |  |  | Urogynecology |
|  | Change from baseline OABSS ‐ daytime frequency score: ‐0.45 vs. ‐ | Withdrawal due to adverse events: 2/40 (5.0%) | and Clinical |
|  | 0.48 | vs. 5/40 (12.5%) | Pathology |
| N = 80 | Change from baseline OABSS ‐ urgency score: ‐1.45 vs. ‐1.35 | Serious adverse events: NR |  |
| Italy | Change from baseline OABSS ‐ urge incontinence score: ‐0.97 vs. ‐ |  |  |
|  | 1.21 |  |  |
| (Fair) |  |  |  |

| **Author, Year Total Population (Included population) Country**  **Trial name (Quality rating)** | **Population** | **Interventions** | **Age, y Gender,**  **% Female Race** | **Other population characteristics**  ***(prognostic)*** |
| --- | --- | --- | --- | --- |
| Yamaguchi et al., | Adults with | Mirabegron 50 mg (n = 380) | Age: 58.3 | Mean weight, kg: 56 |
| 2014 [20] | OAB | vs. |  |  |
|  | symptoms | Tolterodine 4 mg (n = 378) | Female: | Duration of OAB symptoms, months: |
|  | for >24 |  | 83.4% | 72.8 |
| N = 758 | weeks | Duration: 12 weeks |  |  |
|  |  |  | Race: NR | Previous OAB drug therapy, %: 35.8% |
| Japan |  |  |  |  |
|  |  |  |  | Type of OAB, %: |
| (Fair) |  |  |  | ‐No incontinence: 9.5% |
|  |  |  |  | ‐Urgency incontinence: 63.1% |
|  |  |  |  | ‐Mixed stress/urgency incontinence: |
|  |  |  |  | 27.4% |
|  |  |  |  | Mean incontinence episodes /24 h: |
|  |  |  |  | 1.94 |
|  |  |  |  | Mean micturitions /24 h: 11.1 |
|  |  |  |  | Mean urgency (grade ≥3) episodes /24 |
|  |  |  |  | h: 4.2 |
|  |  |  |  | Mean urgency incontinence episodes |
|  |  |  |  | /24 h: 1.75 |

| **Author, Year Total Population (Included population) Country**  **Trial name (Quality rating)** | **Efficacy/effectiveness outcomes**  ***(from PICOS; see checklist)*** | **Harms**  ***(from PICOS; see checklist)*** | **Funding/ Comments** |
| --- | --- | --- | --- |
| Yamaguchi et al., | Mirabegron 50 mg vs. Tolterodine 4 mg | Mirabegron 50 mg vs. Tolterodine 4 mg | Astellas |
| 2014 [20] |  |  | Pharmaceuticals |
|  | Mean change from baseline number of incontinence episodes /24 | Withdrawal due to adverse events: 15/380 |  |
|  | h (SD): ‐1.12 (1.48) vs. ‐0.97 (1.61) | (3.9%) vs. 13/378 (3.4%); RR 1.15 (95% CI 0.55 |  |
| N = 758 |  | to 2.38) |  |
|  | Mean change from baseline number of micturitions /24 h (SD): ‐ |  |  |
| Japan | 1.67 (2.21) vs. ‐1.40 (2.18) | Serious adverse events: 3/379 (0.8%) vs. 4/375 |  |
|  |  | (1.1%) |  |
| (Fair) | Mean change from baseline number of urgency episodes (grade |  |  |
|  | ≥3) /24 h (SD): ‐1.85 (2.56) vs. ‐1.66 (2.56)  Zero incontinence for 3 days per diary (%): 172/338 (50.9) vs. 161/329 (48.9) | Specific adverse events: |  |
|  |  | ‐ Constipation: 13/379 (3.4%) vs. |  |
|  |  | 13/375(3.5%); RR 0.99 (95% CI 0.46 to 2.11) |  |
|  |  | ‐Dry mouth: 10/379 (2.6%) vs. 50/375 (13.3%); |  |
|  |  | RR 0.20 (95% CI 0.10 to 0.38) |  |
|  |  | ‐Tachycardia: 1/379 (0.3%) vs. 0/375 (0%) |  |

**Table S5. Quality assessment of included studies published after 2012 review**

| **Author, Year**  **Study name** | **Randomization adequate?** | **Allocation concealment adequate?** | **Groups similar at baseline?** | **Clinician blinded?** | **Patient blinded?** | **Intention to treat?** | **Acceptable level of overall attrition (≤20%)?** | **Acceptable level of differential attrition (<10%)?** | **Overall quality** |
| --- | --- | --- | --- | --- | --- | --- | --- | --- | --- |
| Abrams, 2017^1^  (SYMPHONY) | Unclear | Unclear | Yes | Yes | Yes | No | Yes | Unclear | Fair |
| Aziminekoo, 2014^2^  Jafarabadi, 2015a^3^ | Unclear | Unclear | Unclear (not shown) | Unclear | Unclear | Unclear | Unclear | Unclear | Poor |
| Batista, 2015^4^  (BEYOND) | Yes | Yes | Yes | Yes | Yes | Yes (2% missing) | Yes | Yes | Good |
| But, 2012^5^ | Yes | Unclear | Yes for OAB outcomes at baseline, unclear for others | No, open- label | No, open- label | No, 19.7% missing | Yes | Yes | Poor |
| Chapple, 2013a^6^  Astellas, 2010^7^  (DRAGON) | Unclear | Unclear | Unclear | Yes | Yes | Yes | Yes | Unclear | Fair |
| Chapple, 2013b^8^  (TAURUS) | Yes | Unclear | Yes | Yes | Yes | Yes | No; 22.7% | Yes | Fair |
| Dede, 2013^9^ | No; "picking one envelope" | Yes; opaque sealed envelopes | No; potentially not incontinence and menopause | Unclear | Unclear | Unclear | Unclear | Unclear | Poor |
| Drake, 2016^10^  (BESIDE) | Yes’ “interactive response technology” | Yes; “double dummy packaging” | Yes | Yes | Yes | Yes | Yes | Yes | Good |

| **Author, Year**  **Study name** | **Randomization adequate?** | **Allocation concealment adequate?** | **Groups similar at baseline?** | **Clinician blinded?** | **Patient blinded?** | **Intention to treat?** | **Acceptable level of overall attrition (≤20%)?** | **Acceptable level of differential attrition (<10%)?** | **Overall quality** |
| --- | --- | --- | --- | --- | --- | --- | --- | --- | --- |
| Ercan, 2015^11^ | Yes; web-based | Unclear; likely yes (web based) | Yes | Unclear | Unclear | Unclear | Unclear | Unclear | Fair |
| Gratzke, 2018^12^  (SYNERGY II) | Yes; interactive response technology system | Yes | Yes | Yes | Yes | Yes | Yes | Yes | Good |
| Herschorn, 2017^13^  Robinson, 2017^14^  White, 2018^15^  (SYNERGY) | Yes | Unclear | Yes | Unclear, noted as double blind | Unclear, noted as double blind | No; 6% excluded from full analysis set | Yes | Yes | Fair |
| Jafarabadi, 2015b^16^ | Yes; random numbers table | Unclear | Yes | Unclear; described as double blind | Unclear; described as double blind | Unclear | Yes | Yes | Fair |
| Khullar, 2013^17^ Khullar, 2016^18^  (SCORPIO) | Yes | Yes | Yes | Yes, double-dummy | Yes, double-dummy | Yes | Yes | Yes | Good |
| Kinjo, 2016^19^ | No; odd or even birth months | Unclear | Yes | Unclear | Unclear | Unclear | Unclear | Unclear | Poor |
| Kosilov, 2015^20^ | Yes | Unclear | Unclear | Unclear | Unclear | Unclear | Unclear | Unclear | Poor |
| Kuo, 2015^21^ | Yes | Unclear | Yes | Unclear; described as double blind | Unclear; described as double blind | Yes | Yes | Yes | Fair |

| **Author, Year**  **Study name** | **Randomization adequate?** | **Allocation concealment adequate?** | **Groups similar at baseline?** | **Clinician blinded?** | **Patient blinded?** | **Intention to treat?** | **Acceptable level of overall attrition (≤20%)?** | **Acceptable level of differential attrition (<10%)?** | **Overall quality** |
| --- | --- | --- | --- | --- | --- | --- | --- | --- | --- |
| Manjunatha, 2015^22^ | Yes | Unclear | Mostly yes, difference in frequency (reported as 1.0 vs 0.5) | No, open-label | No, open-label | Yes | Yes | Yes | Fair |
| Rana, 2016^23^ | Unclear | Unclear | Unclear | Unclear | Unclear | Unclear | Unclear | Unclear | Poor |
| Staskin, 2018^24^  Herschorn, 2018^25^  (PREFER) | Yes | Yes | No; fewer older patients and less incontinence in tolterodine group | Yes | Yes | Yes | Yes | Yes | Fair |
| Vecchioli Scaldazza, 2016^26^ | Yes | Yes | Yes for OAB outcomes at baseline, unclear for others | No, open- label | No, open- label | No, 25% missing | No, 25%  missing | Yes | Fair |
| Yamaguchi, 2014^27^ | Unclear | Unclear | Yes | No | Yes | Yes | Yes | Yes | Fair |

**Table S6. Specific adverse events of included studies published after 2012 review**

| Pharmacological Interventions | # of studies (number of patients) | Blurred Vision, n/N (%) | | Cardiac Arrhythmias, n/N (%) | | Constipation, n/N (%) | | Dizziness, n/N (%) | | Dry Mouth, n/N (%) | | Falls/ Syncope, n/N (%) | |
| --- | --- | --- | --- | --- | --- | --- | --- | --- | --- | --- | --- | --- | --- |
|  |  | Combination | Mirabegron | Combination | Mirabegron | Combination | Mirabegron | Combination | Mirabegron | Combination | Mirabegron | Combination | Mirabegron |
| Mirabegron 25 mg plus solifenacin 5 mg vs. mirabegron 25 mg | 2 (n = 1,497) | 7/997 (0.70) | 1/500 (0.20) | NR | NR | 42/997 (4.21) | 6/500 (1.20) | 1/144 (0.69)‡ | 1/77 (1.30)‡ | 95/997 (9.53) | 19/500 (3.80) | NR | NR |
| Mirabegron 50 mg plus solifenacin 5 mg vs. mirabegron 25 mg | 2 (n = 1,501) | 8/1,001 (0.80) | 1/500 (0.20) | NR | NR | 33/1,001 (3.30) | 6/500 (1.20) | 0/153 (0.0)‡ | 1/77 (1.30)‡ | 81/1,001 (8.09) | 19/500 (3.80) | NR | NR |
| Mirabegron 25 mg plus solifenacin 5 mg vs. mirabegron 50 mg | 2 (n = 1,497) | 7/997 (0.70) | 1/500 (0.20) | NR | NR | 42/997 (4.21) | 14/500 (2.80) | 1/144 (0.69) | 0/78 (0.0) | 95/997 (9.53) | 18/500 (3.60) | NR | NR |
| Mirabegron 50 mg plus solifenacin 5 mg vs. mirabegron 50 mg | 3 (n = 3,012) | 8/1,001 (0.80)† | 1/500 (0.20)† | NR | NR | 73/2,207 (3.31) | 17/805 (2.11) | 13/1,359 (0.96)† | 4/383 (1.04)† | 155/2,207 (7.02) | 30/805 (3.73) | NR | NR |
|  |  | Combination | Solifenacin | Combination | Solifenacin | Combination | Solifenacin | Combination | Solifenacin | Combination | Solifenacin | Combination | Solifenacin |
| Mirabegron 25 mg plus solifenacin 5 mg vs. solifenacin 5 mg | 2 (n = 1,420) | 7/997 (0.70) | 2/588 (0.34) | NR | NR | 42/997 (4.21) | 12/588 (2.04) | 1/144 (0.69)‡ | 1/156 (0.64)‡ | 95/997 (9.53) | 43/588 (7.31) | NR | NR |
| Mirabegron 50 mg plus solifenacin 5 mg vs. solifenacin 5 mg | 4 (n = 4,542) | 8/1,001 (0.80)† | 2/579 (0.34)† | NR | NR | 106/2932 (3.62) | 41/1,610 (2.55) | 19/2,084 (0.91)* | 12/1,187 (1.01)* | 198/2,932 (6.75) | 102/1,610 (6.34) | NR | NR |
| Mirabegron 25 mg plus solifenacin 5 mg vs. solifenacin 10 mg | 1 (n = 222) | 2/144 (1.39) | 1/78 (1.28) | NR | NR | 4/144 (2.78) | 2/78 (2.56) | 1/144 (0.69) | 3/78 (3.85) | 21/144 (14.58) | 23/78 (29.49) | NR | NR |
| Mirabegron 50 mg plus solifenacin 5 mg vs. solifenacin 10 mg | 2 (n = 1,675) | 2/153‡ | 1/78‡ | NR | NR | 11/878 (1.25) | 35/797 (4.39) | 6/878 (0.68) | 11/797 (1.38) | 36/878 (4.10) | 63/797 (7.90) | NR | NR |
|  |  | Mirabegron | Solifenacin | Mirabegron | Solifenacin | Mirabegron | Solifenacin | Mirabegron | Solifenacin | Mirabegron | Solifenacin | Mirabegron | Solifenacin |
| Mirabegron 25 mg vs. Solifenacin 5 mg | 2 (n = 1,077) | 1/498 (0.20) | 2/579 (0.35) | NR | NR | 6/498 (1.20) | 9/579 (1.55) | 1/75 (1.33)‡ | 1/156 (0.64)‡ | 19/498 (3.82) | 43/579 (7.43) | NR | NR |
| Mirabegron 50 mg vs. solifenacin 5 mg | 4 (n = 3,557) | 7/1436 (0.49)* | 6/1513 (0.40)* | NR | NR | 38/1741 (2.18) | 39/1816 (2.15) | 5/383 (1.31)† | 1/459 (0.22)† | 59/1741 (3.39) | 115/1816 (6.33) | NR | NR |
| Mirabegron 25 mg vs. solifenacin 10 mg | 1 (n = 155) | 0/77 (0) | 1/78 (1.28) | NR | NR | 0/77 (0) | 4/78 (5.13) | 0/77 (0) | 3/78 (3.85) | 2/77 (2.60) | 23/78 (29.49) | NR | NR |
| Mirabegron 50 mg vs. solifenacin 10 mg | 1 (n = 156) | 1/78 (1.28) | 1/78 (1.28) | NR | NR | 3/78 (3.85) | 4/78 (5.13) | 0/78 (0) | 3/78 (3.85) | 4/78 (5.13) | 23/78 (29.49) | NR | NR |
|  |  | Mirabegron | Tolterodine | Mirabegron | Tolterodine | Mirabegron | Tolterodine | Mirabegron | Tolterodine | Mirabegron | Tolterodine | Mirabegron | Tolterodine |
| Mirabegron 50 mg vs. tolterodine 4 mg | 6 (n = 5,001) | 12/319 (3.76)‡ | 11/325 (3.38)‡ | 57/1,990 (2.86)⁑ | 78/2,003 (3.89)⁑ | 74/2,538 (2.92) | 75/2,463 (3.05) | 33/1,347 (2.45)* | 29/1,268 (2.29)* | 97/2,538 (3.82) | 256/2,463 (10.39) | 2/1,178 (0.17)† | 1/1,183 (0.08)† |
|  |  | Solifenacin | Darifenacin | Solifenacin | Darifenacin | Solifenacin | Darifenacin | Solifenacin | Darifenacin | Solifenacin | Darifenacin | Solifenacin | Darifenacin |
| Solifenacin 5 mg vs. darifenacin 7.5 mg | 1 (n = 61) | 10/32 (31.25) | 9/29 (31.03) | NR | NR | 8/32 (25.0) | 8/29 (25.59) | 7/32 (21.88) | 4/29 (13.79) | 13/32 (40.63) | 18/29 (62.07) | NR | NR |
|  |  | Solifenacin | Fesoterodine | Solifenacin | Fesoterodine | Solifenacin | Fesoterodine | Solifenacin | Fesoterodine | Solifenacin | Fesoterodine | Solifenacin | Fesoterodine |
| Solifenacin 5 mg vs. fesoterodine 4 mg | 1 (n = 119) | NR | NR | NR | NR | 1/60 (1.67) | 3/59 (5.08) | NR | NR | 3/60 (5.0) | 8/59 (13.56) | NR | NR |
|  |  | Fesoterodine | Tolterodine | Fesoterodine | Tolterodine | Fesoterodine | Tolterodine | Fesoterodine | Tolterodine | Fesoterodine | Tolterodine | Fesoterodine | Tolterodine |
| Fesoterodine 8 mg vs. tolterodine ER 4 mg | 3 (n = 3,873) | NR | NR | NR | NR | 92/1926 (4.78) | 66/1947 (3.39) | 18/1926 (0.93) | 14/1947 (0.72) | 551/1926 (28.60) | 291/1947 (14.95) | NR | NR |
| Fesoterodine 4 mg vs. tolterodine 4 mg | 1 (n = 562) | NR | NR | NR | NR | 9/272 (3.31) | 8/290 (2.76) | 4/272 (1.47) | 4/290 (1.38) | 59/272 (21.69) | 49/290 (16.90) | NR | NR |
|  |  | Tolterodine | Oxybutynin | Tolterodine | Oxybutynin | Tolterodine | Oxybutynin | Tolterodine | Oxybutynin | Tolterodine | Oxybutynin | Tolterodine | Oxybutynin |
| Tolterodine 4 mg vs. oxybutynin 15 mg | 4 (n = 639) | NR | NR | NR | NR | NR | NR | NR | NR | 140/336 (41.67) | 242/303 (79.87) | NR | NR |
| Tolterodine ER/IR 4 mg vs. oxybutynin 10 mg | 2 (n = 1,168) | 2/193 (1.04)‡ | 4/185 (2.16)‡ | NR | NR | 43/592 (7.26) | 38/576 (6.60) | 8/193 (4.15)‡ | 9/185 (4.86)‡ | 153/592 (25.84) | 168/576 (29.17) | NR | NR |
|  |  | Solifenacin | Tolterodine | Solifenacin | Tolterodine | Solifenacin | Tolterodine | Solifenacin | Tolterodine | Solifenacin | Tolterodine | Solifenacin | Tolterodine |
| Solifenacin 5 mg vs. tolterodine 4 mg | 5, (n = 2,067) | 28/1,027 (2.73)⁑ | 25/1,025 (2.44)⁑ | NR | NR | 38/1,066 (3.56) | 12/1,061 (1.13) | 1/39 (2.56)‡ | 0/36 (0.0)‡ | 60/473 (12.68) | 89/454 (19.60) | NR | NR |
| Solifenacin 10 mg vs. tolterodine 4 mg | 3, (n = 839) | 39/421 (9.26) | 16/418 (3.83) | NR | NR | 40/409 (9.78) | 11/406 (2.71) | NR | NR | 85/415 (20.48) | 80/412 (19.42) | NR | NR |
|  |  | Solifenacin | Oxybutynin | Solifenacin | Oxybutynin | Solifenacin | Oxybutynin | Solifenacin | Oxybutynin | Solifenacin | Oxybutynin | Solifenacin | Oxybutynin |
| Solifenacin 10 mg vs. oxybutynin 15 mg | 1, (n = 132) | 2/68 (2.94) | 0/64 (0.0) | NR | NR | 9/68 (13.24) | 4/64 (6.25) | NR | NR | 24/68 (35.29) | 53/64 (82.81) | NR | NR |
|  |  | Darifenacin | Tolterodine | Darifenacin | Tolterodine | Darifenacin | Tolterodine | Darifenacin | Tolterodine | Darifenacin | Tolterodine | Darifenacin | Tolterodine |
| Darifenacin 15 mg vs. tolterodine 4 mg | 1, (n = 335) | NR | NR | NR | NR | 28/112 (25.0) | 28/223 (12.6) | NR | NR | 28/112 (25.0) | 59/223 (26.5) | NR | NR |

*Adverse events with k = 3 studies

⁑Adverse events with k = 4 studies

†Adverse events with k = 2 studies

‡Adverse events with k = 1 studies

Note: The Cochrane review included one study abstract for the tolterodine vs. trospium that did not contain adverse event data. One newly identified study comparing trospium with darifenacin did not contain useable adverse event data.
